# Cellular senescence‐related long noncoding ribonucleic acids: Predicting prognosis in hepatocellular carcinoma

**DOI:** 10.1002/cnr2.1791

**Published:** 2023-02-01

**Authors:** Hao Huang, Hao Yao, Yaqing Wei, Ming Chen, Jinjin Sun

**Affiliations:** ^1^ Department of Hepatopancreatobiliary Surgery The Second Hospital of Tianjin Medical University Tianjin China

**Keywords:** cellular senescence, hepatocellular carcinoma, long noncoding ribonucleic acids, prognostic model

## Abstract

**Background:**

Due to their inherent role in cell function, long non‐coding ribonucleic acids (lncRNAs) mediate changes in the microenvironment, and thereby participate in the development of cellular senescence.

**Aims:**

This study aimed to identify cellular senescence‐related lncRNAs that could predict the prognosis of liver cancer.

**Methods and Results:**

Gene expression and clinical data were downloaded from the UCSC Xena platform, ICGC, and TCGA databases. Cox regression and LASSO regression were used to establish a cellular senescence‐related lncRNA model. ROC curves and Kaplan–Meier survival curves were then constructed to predict patient prognosis. Cox regression analysis and clinical characteristics were used to evaluate the capability of the model. Tumor mutational burden and tumor‐infiltrating immune cell analyses were subsequently performed in the risk subgroups and the samples in the entire cohort were reclustered. Finally, potential small molecule immune‐targeted drugs were identified based on the model. The cellular senescence‐related prognostic model that was constructed based on *AGAP11* and *FAM182B*. Along with the results of Cox regression and Lasso regression, the risk score was found to be an independent factor for predicting overall survival in cohorts. In the subgroup analysis, the prognosis of the low‐risk group in each cohort was significantly higher than that of the high‐risk group; the area under temporal ROC curves and clinical ROC curves were all greater than 0.65, respectively. C‐index shows that the risk scores are greater than 0.6, showing the stability of the model. The high‐risk group demonstrated lower tumor microenvironment and higher tumor mutational burden scores, further verifying the reliability of the model grouping results. Analysis of tumor‐infiltrating immune cells indicated that CD8+ and γδ T cells were more abundant among patients in the low‐risk group; cluster reorganization indicated that the two groups had different prognoses and proportions of immune cells. The *p* value of potential drugs predicted based on the expression of model lncRNAs were all less than .05, demonstrating the potential of model lncRNAs as therapeutic targets to some extent.

**Conclusion:**

A prognostic model based on cellular senescence‐associated lncRNAs was established and this may be used as a potential biomarker for the prognosis assessment of liver cancer patients.

## 1 INTRODUCTION

Hepatocellular carcinoma (HCC) is the third leading cause of global cancer‐related deaths and is a highly devastating malignancy; it has a low five‐year survival rate of approximately 18%.^1^, ^2^ Owing to the absence of obvious symptoms in the early stages of HCC, many patients are only diagnosed in the advanced stages of this malignancy.^3^ As a common solid tumor, the tumor microenvironment (TME) of HCC is characterized by immune suppression, which is caused by various mechanisms including the recruitment of immune suppressive cells, a reduction in antitumor effector cells, changes in cytokine levels, and an increase in immune checkpoint proteins; these mechanisms are interactive.^4^ Immunotherapy is therefore likely to have considerable potential in the treatment of HCC.

Senescence is a state in which cells are alive and biologically active for an extended period of time without cell division; they eventually resume proliferation, while sending heterotypic signals to their microenvironment.^5^ The transition of cells to a senescent state or death (via apoptosis) may be induced by internal factors (deoxyribonucleic acid damage, oxidative stress, and metabolic system disturbances) or external “commands” (transmitted mainly through receptors), which affect the internal balance of signaling pathways. External signals may originate from either the immediate external environment (possibly from immune cells, as part of their function) or senescent cells (with respect to a senescence‐associated secretory phenotype). These signals may force surrounding cells to senesce; this in turn promotes local inflammation and attracts immune cells.^6^ Systemic regulators of the immune response have been shown to participate in mechanisms related to the cell cycle and aging; this provides a theoretical basis for investigating the relationship between the immune system, aging mechanisms, and cancer.^7^

Long non‐coding ribonucleic acids (lncRNAs) are noncoding RNAs of >200 nucleotides that lack open reading frame coding potential and have an array of roles in various biological processes, including epigenetics, transcription, and post‐transcriptional regulation of gene expression.^8^ In the process of tumorigenesis, lncRNAs are involved in the regulation of tumor suppressor genes and oncogene expression. As they are directly involved in the epigenetic modification of genes, and affect the regulation of microRNAs, lncRNAs may be used as potential biomarkers for the early diagnosis and prognostication of cancer.^9^, ^10^, ^11^, ^12^, ^13^ Technological developments mean lncRNAs may be conveniently stored, acquired, screened, and detected using less invasive methods; therefore, they offer certain advantages as cancer biomarkers.^14^

In this study, we used bioinformatics technology to establish a prognostic model based on cellular senescence‐related lncRNAs and showed that the model could predict prognosis in patients with HCC. We collected liver cancer samples from the database, identified cellular senescence‐associated differentially expressed genes using bioinformatics techniques, explored their characteristics in the TME and tumor‐infiltrating immune cells, and reclustered them into groups based on gene correlations. Finally, small molecule immune‐targeted drugs were identified based on the lncRNA‐based model.

## 2 MATERIALS AND METHODS

### 2.1 Acquisition of liver cancer sample data

Gene expression and clinical data of patients were obtained from the UCSC Xena (https://xenabrowser.net/datapages/), the Cancer Genome Atlas (TCGA) Liver Hepatocellular Carcinoma (https://portal.gdc.cancer.gov/), and International Cancer Genome Consortium (ICGC) Liver Cancer‐RIKEN, Japan databases (https://dcc.icgc.org/). We use the Strawberry Perl software and tidyverse R software package to integrate gene expression data in the “TCGA‐GTEX‐Gene‐Counts” file downloaded from the UCSC Xena database and the “LIRI‐JP” file downloaded from the ICGC database. Differentially expressed genes were identified among normal and tumor samples (*p* < .05, FDR < 0.05, and $\left|\log2\mathrm{FC}\right|>1.0$) based on transcriptomic data analysis results obtained by the DESeq2 package. According to the differentially expressed genes, the “TCGA‐LIHC” data in TCGA database and the “LIRI‐JP” data in the ICGC database were integrated to generate an FPKM gene matrix for further analysis.

### 2.2 Identification of cellular senescence‐associated lncRNAs

We logged onto the GeneCards website of the human gene database (https://www.genecards.org/), and used “cellular senescence” as the keyword withgift score > 10.0 and relevance score > 4.0 as screening criteria to determine the cellular senescence genome. Subsequently, we took the intersection of cellular senescence genome and FPKM gene matrix, and analyze the intersection genes with the limma R package (*p* < .05, FDR < 0.05, and $\left|\log2\mathrm{FC}\right|>1.0$) to identify the differentially expressed cellular senescence genes and draw a volcano map. Finally, we use the Strawberry Perl software and limma R package to perform a Pearson correlation analysis on the differentially expressed cellular senescence genes and lncRNAs to determine cellular senescence‐related lncRNAs (PCCs > 0.4 and *p* < .001) and draw a network diagram.

### 2.3 Establishing the risk signature

To exclude patient deaths that may have been caused by factors such as surgical stress or postoperative complications, this analysis excluded 52 tumor samples (562 tumor samples retained) with missing overall survival (OS) data or OS ≤30 days. Combined with the OS and FPKM gene matrix of the tumor samples, a univariate Cox (Uni‐Cox) regression analysis was used to screen survival‐related lncRNAs from the cellular senescence‐related lncRNAs (*p* < .05), and the results were drawn into a forest plot. Results of the Uni‐Cox regression analysis were then subjected to least absolute shrinkage and selection operator (LASSO) regression using 10‐fold cross‐validation and a *p*‐value of .05, which was set to perform 1000 iterations, with 1000 random stimuli for each iteration to prevent overfitting. The LASSO regression results were analyzed using a multivariate Cox (Multi‐Cox) regression to determine which lncRNAs would ultimately be included in the model (*p* < .05) and to draw a forest plot. The risk score was calculated using the following formula:

$$\text{Risk score}=\sum_{k=1}^{n}\text{coef}\left({\text{lncRNA}}^{k}\right)\times \text{expr}\left({\text{lncRNA}}^{k}\right).$$

In the equation, coef (lncRNAn) denotes the coefficient of lncRNAs that correlated with survival, and expr (lncRNAn) denotes lncRNA expression. Low‐ and high‐risk subgroups were established based on the median risk score. Finally, to reduce statistical bias in this analysis and further verify the stability of the model, we randomly divided the entire cohort of 562 patients into a training cohort (*N* = 282) and a validation cohort (*N* = 280) at a ratio of 1:1, with similar numbers of patients in low‐ and high‐risk subgroups in each cohort.^15^

### 2.4 Validating the risk signature

To compare the prognosis of low‐ and high‐risk subgroup samples in each cohort, risk score and survival status evaluation and survival analysis curve construction were performed in three independent cohorts (i.e., training, validation, and entire cohort) by implementing the survival, survivor, and phenotmap R packages, respectively. Temporal receiver operating characteristic (ROC) curves of the model at 1, 2, and 3 years were subsequently constructed in three independent cohorts by executing the survival, timeROC, and survminer R packages, respectively. To further verify the effectiveness of the model score, R packages such as survival, caret, and glmnet were used to perform Cox regression analysis to determine whether the risk score and other clinical features were independent variable factors; ROC curves were constructed to compare the accuracy of outcomes predicted by different clinical characteristics. The nomogram including risk scores, gender, and stage was subsequently used to predict patient OS at 3 years. The calibration curves and the concordance index (C‐index) were used to determine matching between the predicted and the actual results. In addition, to observe whether missing samples (60 of 562 samples are missing) have an impact on the cohort, we perform cross‐validation on subgroup clinical characteristics to test cohort independence. Finally, Gene Ontology (GO) and Kyoto Encyclopedia of Genes and Genomes (KEGG) analyses were performed on differentially expressed genes in the low‐ and high‐risk groups using the clusterProfiler, enrichplot, and GOplot R packages (*p* < .05).

### 2.5 Tumor mutation burden analysis

Patients with poorer prognosis are often accompanied by more gene mutations. Therefore, nucleotide mutation data of the TCGA Liver Hepatocellular Carcinoma and ICGC Liver Cancer‐RIKEN, Japan, cohorts were selected for tumor mutation burden (TMB) analysis to observe the differences in TMB values between low‐ and high‐risk subgroups in an entire cohort (62 of 562 samples are missing). Using limma and ggpubr R packages and Strawberry Perl software, samples were divided into low (L)‐TMB and high (H)‐TMB groups (TMB = 3.00), and differences in TMB were compared between samples from the low‐ and high‐risk subgroups; survival curves were then constructed for these groups using the survival and survminer R packages. Furthermore, limma and ggplot2 R packages were used to construct a scatter plot to identify correlation between risk scores and TMB. Finally, survival curves were constructed for the risk subgroups in combination with TMB to further compare the OS of subgroup patients.

### 2.6 Analysis of the TME and tumor‐infiltrating immune cells

To explore differences in the immune microenvironment between low‐ and high‐risk subgroup samples, we analyzed the TME scores and proportion of tumor‐infiltrating immune cells in the subgroups. TME scores were calculated for differentially expressed cellular senescence genes in the low‐ and high‐risk groups using the estimate R package. The preprocessCore and limma R packages were then used to evaluate the proportional difference of tumor‐infiltrating immune cells between the low‐ and high‐risk groups and draw a violin chart (*p* < .05); the CIBERSORT algorithm was used, and Kaplan–Meier survival curves were constructed for the immune cell scores (*p* < .05).

### 2.7 Reclustering model‐related genes

To observe the expression of cellular senescence genes associated with model lncRNAs (*AGAP11* and *FAM182B*) in the samples, the limma and reshape2 R packages were used to perform correlation analysis on model lncRNAs and these genes. Cellular senescence genes associated with the *AGAP11* and *FAM182B* genes were listed in the form of a correlation heatmap (*p* < .05), and the samples were clustered according to these genes using the ConsensusClusterPlus R package. A survival analysis and an analysis of immune composition were then performed on the recluster groups. Finally, a Sankey diagram was plotted for *AGAP11* and *FAM182B* genes versus target genes and presented in boxplots using the ggpubr and limma R packages ($\left|\text{PCCs}>0.4\right|$, *p* < .001).

### 2.8 Potential drug target predictions

To clarify the level of immune escape ability of the samples, immune escape scores were compared between the low‐ and high‐risk groups, which were obtained from the tumor immune dysfunction and exclusion (TIDE) website (http://tide.dfci.harvard.edu/). The pRRophetic R package was used to assess treatment response in the low‐ and high‐risk groups based on half‐maximal inhibitory concentrations of the samples (*p* < .05). Information pertaining to the genes was obtained from the CellMiner website (https://discover.nci.nih.gov/cellminer/home.do), and the potential of model lncRNAs as therapeutic targets was predicted by evaluating the correlation between genes and drug targets (*p* < .05).

## 3 RESULTS

### 3.1 Acquisition and identification of cellular senescence‐associated lncRNAs

**FIGURE 1:**
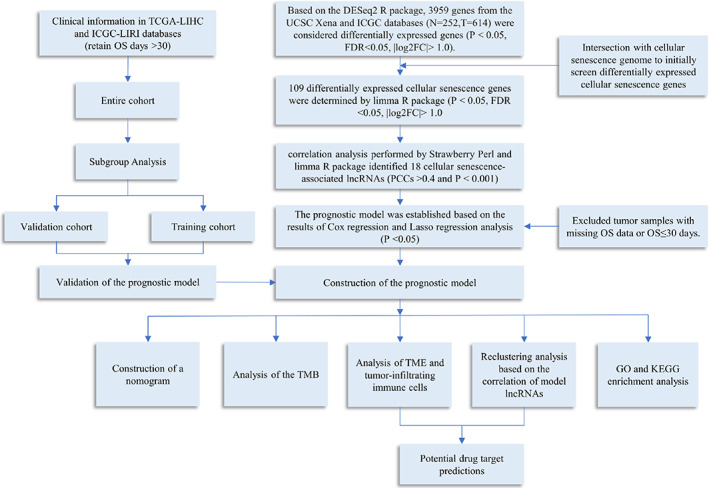
Schematic overview of the study design.

**FIGURE 2:**
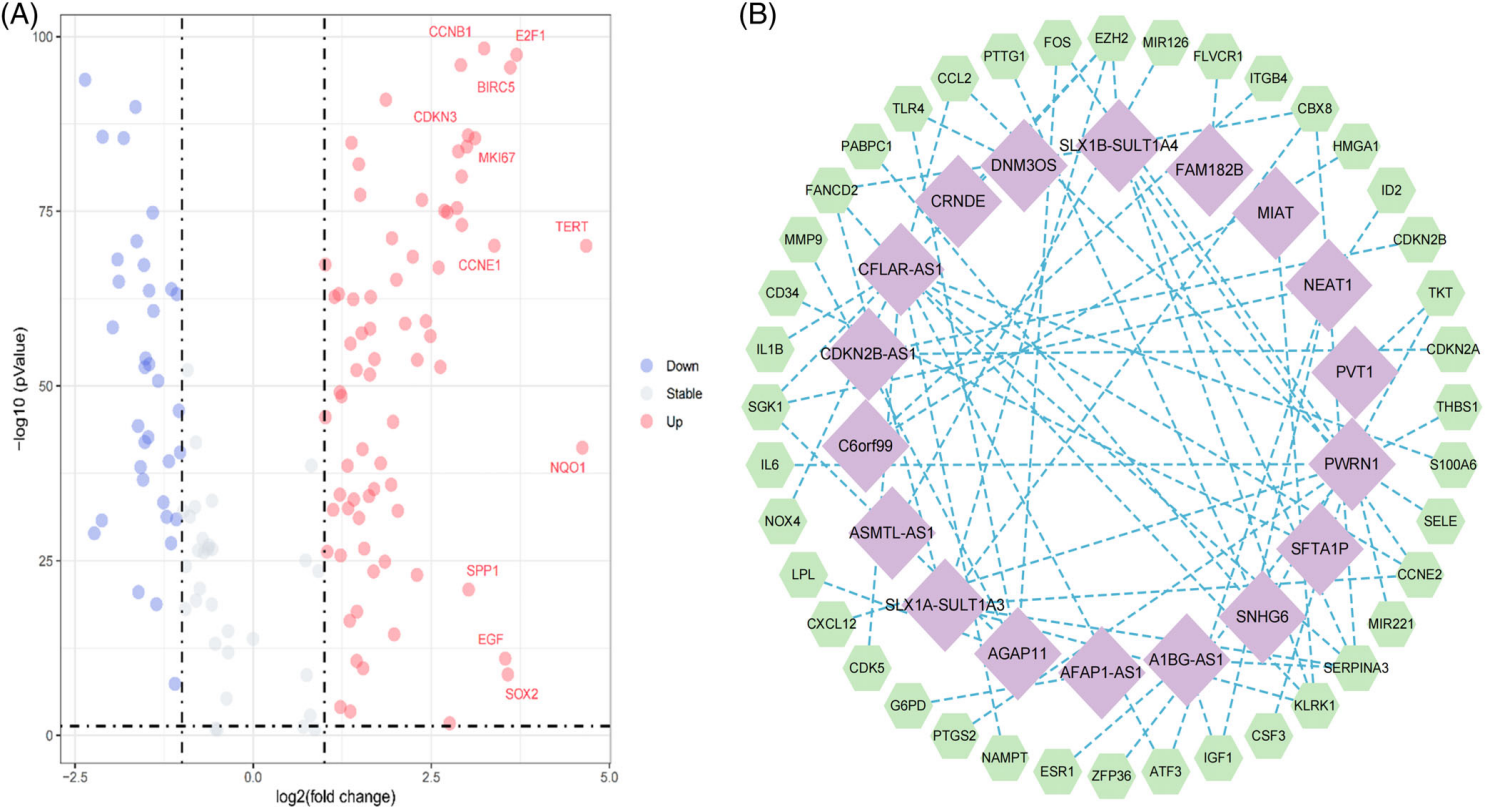
Identification of cellular senescence‐associated lncRNAs. (A) Volcano plot showing differentially expressed cellular senescence genes between tumor and normal tissue samples. (B) Network diagram of differentially expressed cellular senescence genes and lncRNAs. Purple rhombi represent cellular senescence‐associated lncRNAs (PCCs > 0.4, *p* < .001); green hexagons represent differentially expressed cellular senescence genes.

The study flowchart is shown in Figure 1. Transcriptome data for 614 tumor samples and 252 normal tissue samples were obtained and integrated from the UCSC Xena, TCGA, and ICGC databases. The analysis results of Count gene matrix by the DESeq2 R package showed that 3959 genes (including 59 lncRNAs) with $\left|\log2\mathrm{FC}\right|>1.00$, FDR < 0.05, and *p* < .05 were considered differentially expressed genes (Data S1). Using the limma R package to analyze the intersection FPKM gene matrix of differentially expressed genes and cellular senescence genome in normal and tumor samples, 109 differentially expressed cellular senescence genes with $\left|\log2\mathrm{FC}\right|>1.00$, FDR <0.05, and *p* < .05 were identified; 35 of these genes were downregulated, while the others were upregulated (Figure 2A; Data S2). On subsequent correlation analysis of 109 differentially expressed cellular senescence genes, 18 lncRNAs with PCCs > 0.4 and *p* < .001 were selected as cellular senescence‐related lncRNAs for this study (Figure 2B; Data S3).

### 3.2 Construction of the prognostic model

**FIGURE 3:**
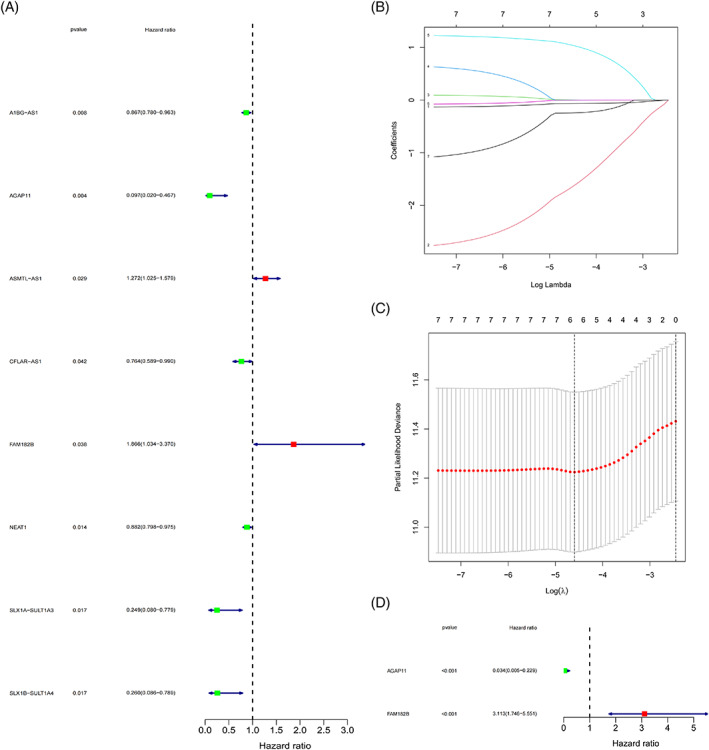
Cox and LASSO regression analyses for determining the model genes. (A) Uni‐Cox regression analysis for identification of prognosis‐associated cellular senescence lncRNAs. (B) 10‐fold cross‐validation of variables in the LASSO regression analysis models. (C) LASSO regression analysis for reducing the number of factors to prevent overfitting. (D) Multi‐Cox regression analysis for determining the model genes.

The Uni‐Cox regression analysis used the bootstrap resampling method based on the optimal risk coefficient and *p*‐value to screen a group of survival‐related lncRNAs from cell senescence‐related lncRNAs (Figure 3A); eight lncRNAs were selected to be associated with OS (*p* < .05). Overfitting of the prognostic signature was avoided by performing a LASSO regression analysis (Figure 3B,C) to further screen for OS‐related lncRNAs when the first‐order value of log (λ) demonstrated the least likelihood of bias (*p* < .05). The Multi‐Cox regression analysis of the LASSO regression results using a bootstrap resampling method identified the best survival‐associated model lncRNAs, including *AGAP11* and *FAM182B* (Figure 3D). Finally, the median risk score calculated from the risk score formula was used to establish low‐risk and high‐risk subgroups (Data S4).

### 3.3 Verification of the prognostic model

**FIGURE 4:**
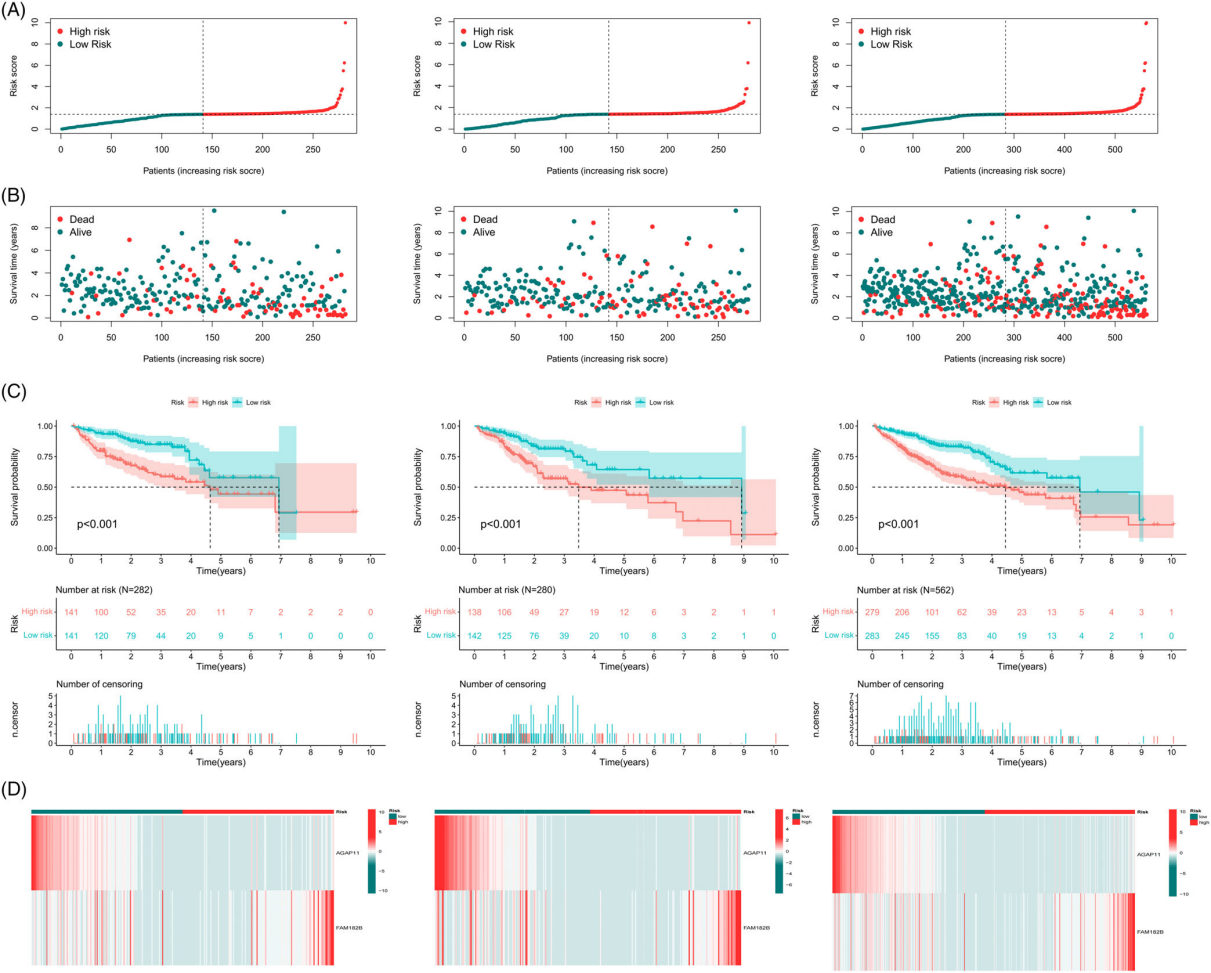
Internal validation of the prognostic model. (A, B) Distribution of risk scores (A) and survival status (B) for risk subgroups in training (left), validation (middle), and entire cohorts (right). (C) Kaplan–Meier survival curves for overall survival among patients in training (left), validation (middle), and entire cohorts (right). (D) Heatmaps of model genes for risk subgroups in training (left), validation (middle), and entire cohorts (right).

**FIGURE 5:**
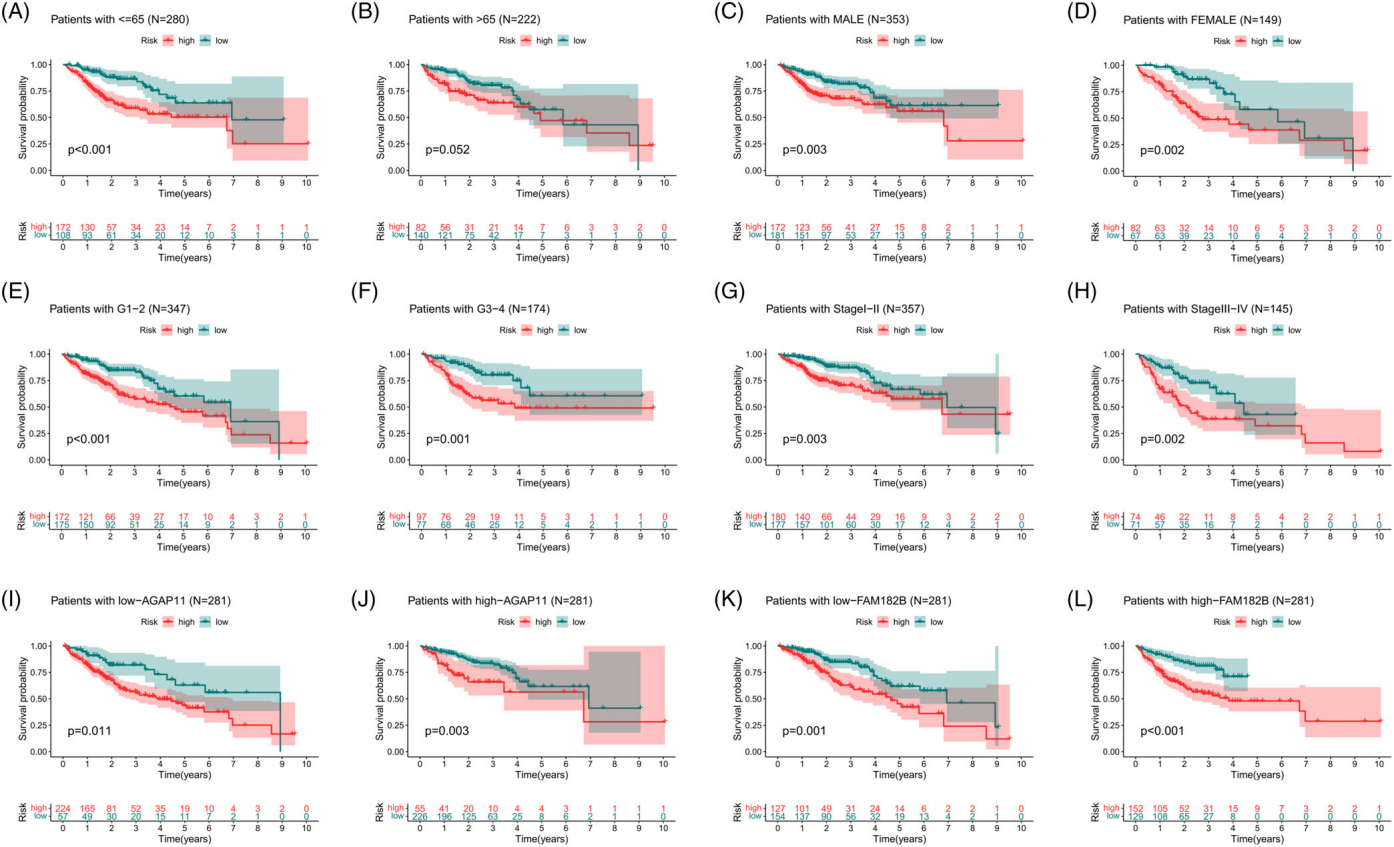
The OS prognostic values of Kaplan–Meier survival curves stratified between low‐ and high‐risk groups of the entire cohort by age (A, B), gender (C, D), tumor grade (E, F), stage of cancer (G, H), and expression level of model lncRNAs (I–L).

Based on subgroup analysis, the entire cohort was divided into training and validation cohorts. In three independent cohorts, the low‐risk group performed better in terms of risk score distribution (Figure 4A), survival status (Figure 4B), and survival time (Figure 4C). The heatmaps show the model gene expression for the low‐risk and high‐risk groups (Figure 4D). Cross‐validation showed that there were no significant differences in clinical characteristics between the cohorts (*p* > .05), proving that the cohorts were independent of each other (Table 1). Kaplan–Meier survival curves based on clinical characteristics of the entire cohort showed statistically significant differences between the low‐ and high‐risk groups (Figure 5A–L).

**TABLE 1:** Internally validated clinical data of the cohort (*N* = 502).

Clinical feature	Type	Training cohort	Validation cohort	Entire cohort	*p*
Age (years)	≤65	137 (54.15%)	143 (57.43%)	280 (55.78%)	.5158
	>65	116 (45.85%)	106 (42.57%)	222 (44.22%)	
Gender	Female	68 (26.88%)	81 (32.53%)	149 (29.68%)	.1976
	Male	185 (73.12%)	168 (67.47%)	353 (70.32%)	
Tumor grade	G1	27 (10.67%)	36 (14.46%)	63 (12.55%)	.2604
	G2	141 (55.73%)	127 (51%)	268 (53.39%)	
	G3	81 (32.02%)	77 (30.92%)	158 (31.47%)	
	G4	4 (1.58%)	9 (3.61%)	13 (2.59%)	
Tumor stage	I	88 (34.78%)	103 (41.37%)	191 (38.05%)	.3166
	II	88 (34.78%)	78 (31.33%)	166 (33.07%)	
	III	72 (28.46%)	60 (24.1%)	132 (26.29%)	
	IV	5 (1.98%)	8 (3.21%)	13 (2.59%)	

### 3.4 Assessment of clinical factors of the prognostic model

**FIGURE 6:**
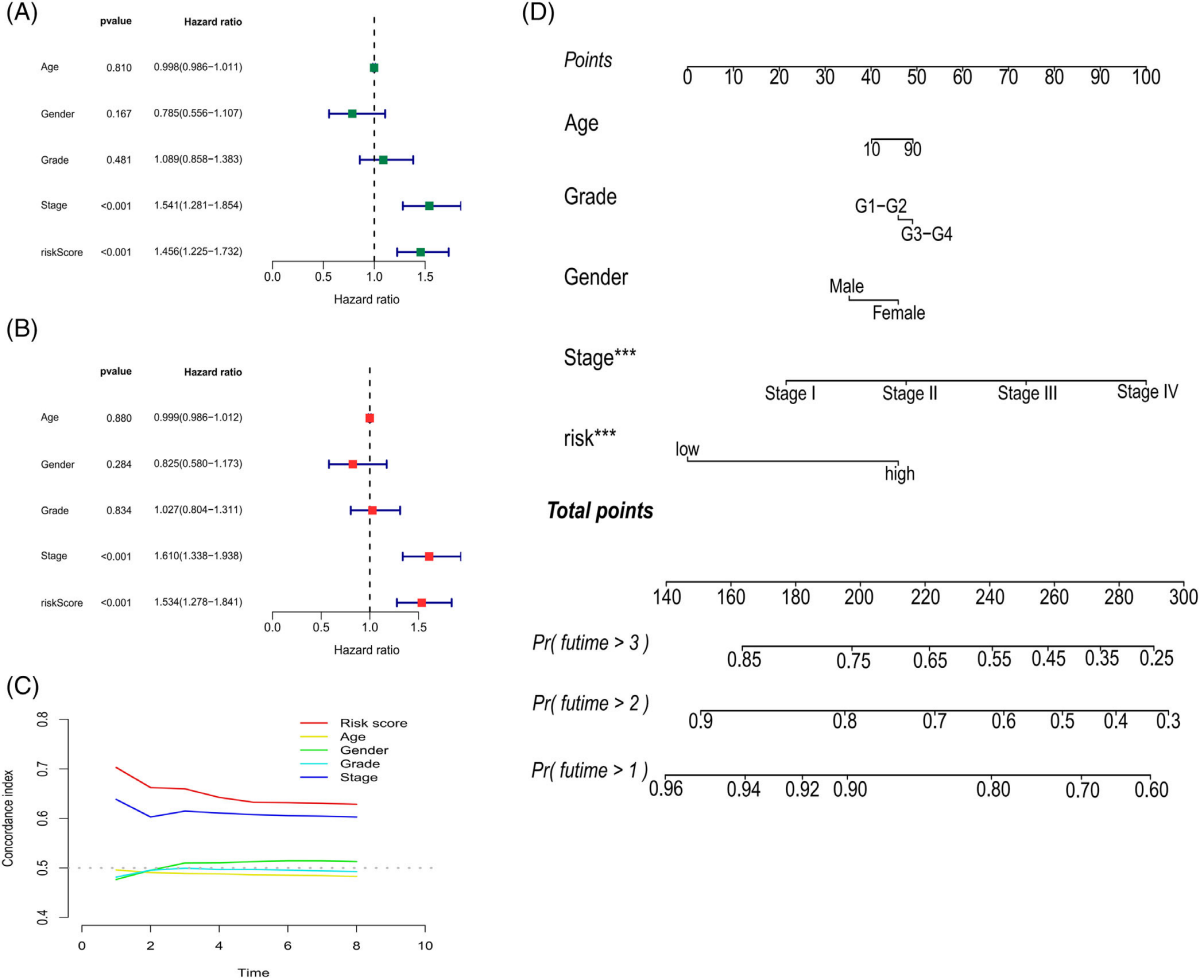
Analysis of independent factors and construction of a nomogram. Forest plots showing hazard ratios and 95% confidence intervals for clinicopathological characteristics in the entire cohort calculated by uni‐Cox (A) and multi‐Cox (B) regression analyses, respectively. (C) The C‐index assessed the predictive ability of each clinical feature. (D) Nomogram based on risk score and other clinical factors for predicting 1‐, 2‐, and 3‐year OS in the entire cohort with liver cancer.

Cox regressions were used to evaluate the clinical characteristics of the entire cohort; in the Uni‐Cox (Figure 6A) and Multi‐Cox regressions (Figure 6B), the hazard ratios for the risk scores were 1.456 and 1.534, respectively, and the *p* value were less than .05, which proved that risk scores could be recognized as an independent risk factor. Notably, except for cases with meaningful risk scores, the *p* value for the tumor stage was <.05. The C‐index (Figure 6C) was >0.6 for both the risk score and tumor stage, which proves that the cellular senescence‐related prognostic model indicated good accuracy and validity in predicting the OS of samples. Finally, a nomogram for the 1–3‐year OS was generated based on the model (Figure 6D).

### 3.5 ROC curves and calibration curve validation of the prognostic model

**FIGURE 7:**
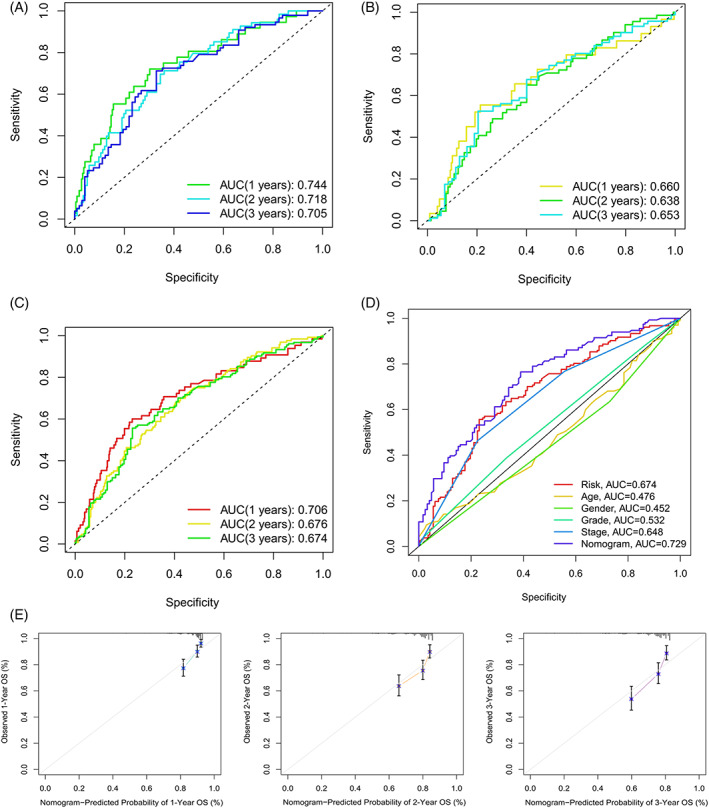
Evaluation of the predictive power of the model. Temporal ROC curves for prediction of patient survival in training (A), validation (B), and entire cohorts (C). (D) ROC curves of clinical characteristics among patients in the entire cohort. (E) Calibration plot of a nomogram for the prognostic model.

Temporal ROC curves were utilized to assess the sensitivity and specificity of the model in predicting prognosis; the area under the curve (AUC) was also calculated. The AUCs for the training cohort (Figure 7A), validation cohort (Figure 7B), and the entire cohort (Figure 7C) were all >0.65, which indicated that the model was reliable in predicting the OS of patients. Clinical ROC curves were also used to evaluate the clinical characteristics of the model; the AUCs for the risk score and total nomogram score were 0.674 and 0.729, respectively, further illustrating the sensitivity of the model (Figure 7D). Finally, verification using calibration curves showed the nomogram to have considerably reliable predictive performance (Figure 7E).

### 3.6 GO and KEGG enrichment analysis of risk subgroups

**FIGURE 8:**
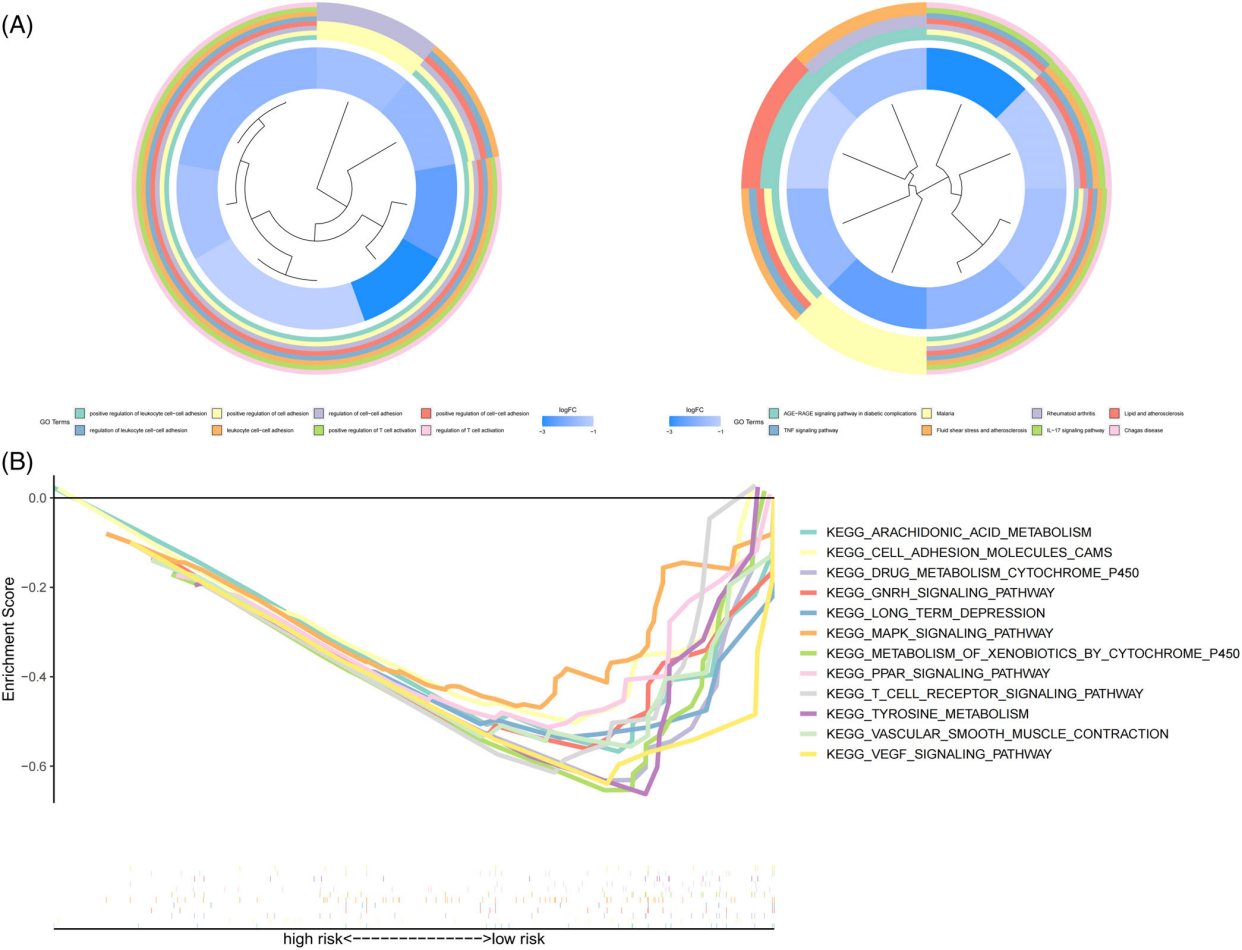
(A): GO and KEGG enrichment analysis. Enrichment plot showing pathways enriched on GO (left) and KEGG (right) analysis. (B) The top 12 pathways of gene set enrichment analysis in the low‐risk group.

To investigate differences in genes between risk subgroups, GO and KEGG analyses were performed on differentially expressed genes (*p* < .05) (Figure 8A), with multiple pathways of GO enrichment possibly related to tumor invasion and immune infiltration; the included pathways positively regulated cell adhesion and T cell activation. A gene set enrichment analysis was then performed for the risk subgroups based on KEGG pathways. In the low‐risk group, 7 of the top 12 pathways (*p* < .05, FDR < 0.25, and $\left|\text{normalized enrichment score}\right|>1.5$) with enrichment showed high correlation with immune infiltration and tumor invasion; these included the vascular endothelial growth factor pathway and the T cell receptor signaling pathways (Figure 8B).

### 3.7 Analysis of TMB in the prognostic model

**FIGURE 9:**
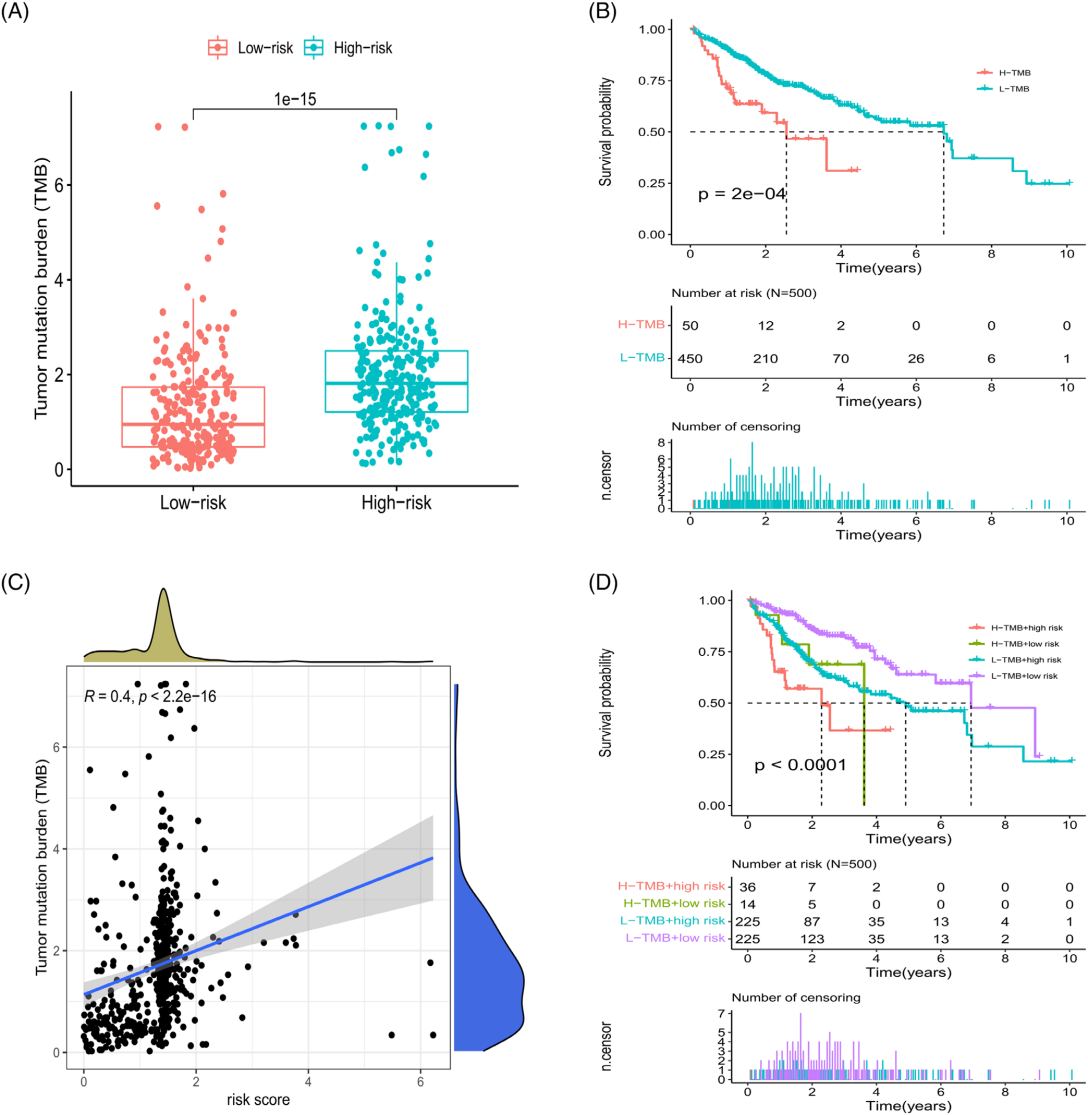
TMB analysis of risk subgroups. (A) Comparison of TMB between the low‐ and high‐risk groups. (B) Kaplan–Meier survival curves of patients in the high‐ and low‐TMB groups (H‐TMB and L‐TMB, respectively), grouped by TMB = 3.00. (C) Scatter plot depicting positive correlation between risk scores and mutation load. (D) Kaplan–Meier survival curves for OS in four patient groups stratified by TMB and risk scores. (Some data time point are missing from the patients' databases).

Calculation of TMB values for the risk subgroups revealed a significantly higher TMB value for the high‐risk group (Figure 9A). The survival curve showed that grouping into L‐TMB and H‐TMB groups impacted OS (Figure 9B); in addition, correlation analysis showed a positive correlation between risk scores and TMB values (Figure 9C). Combining TMB groups with risk subgroups markedly affected survival, with the H‐TMB + high risk group demonstrating significantly poorer prognosis than the L‐TMB + low risk group (Figure 9D). High‐risk subgroups had higher TMB values ​​and shorter OS values. The combined analysis of risk subgroups and TMB values ​​further verified the reliability of the model to a certain extent.

### 3.8 Differences in the TME between risk subgroups

**FIGURE 10:**
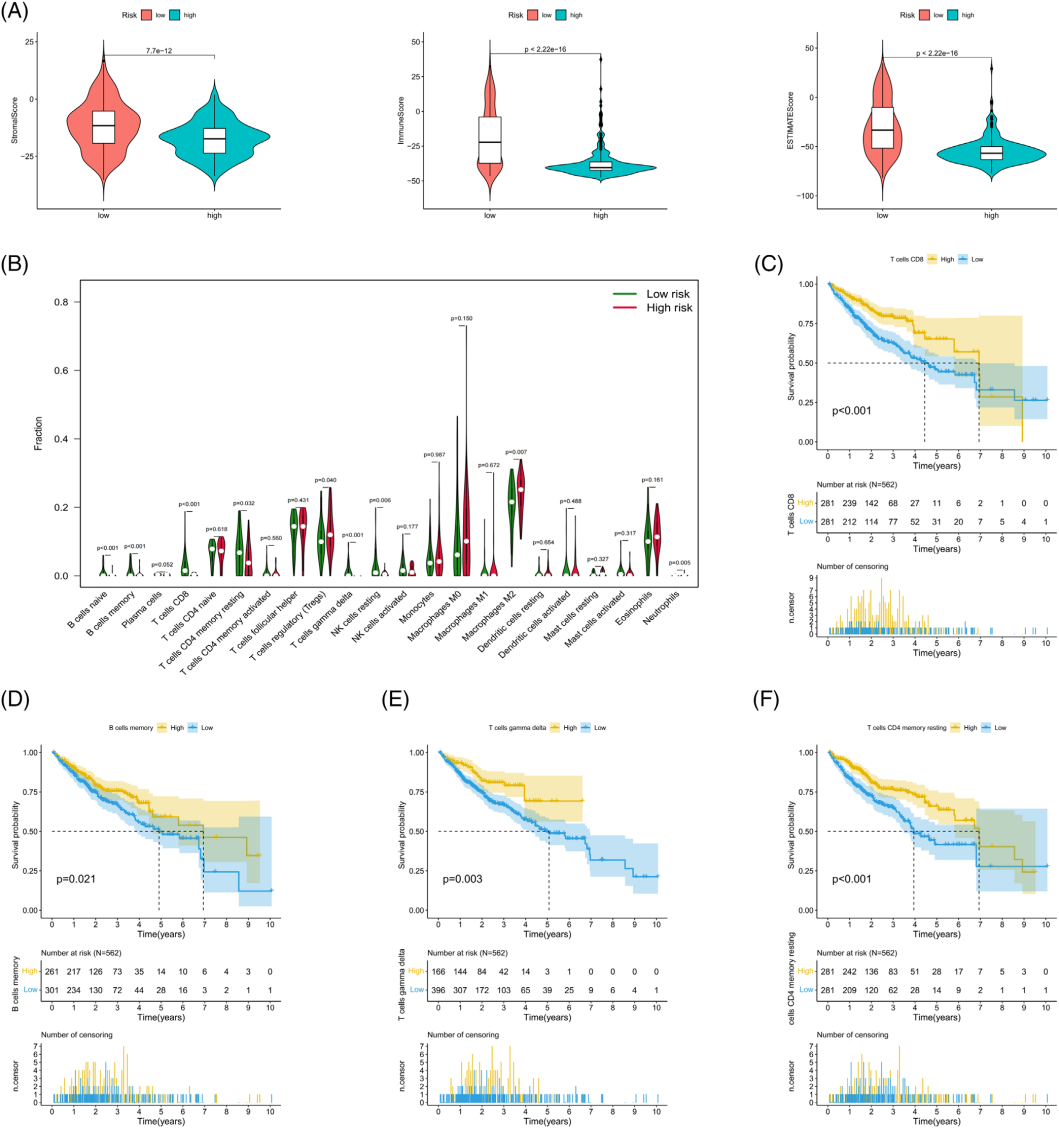
Analysis of the TME for the prognostic model. (A) Boxplots comparing immune stromal scores (left), immune cell scores (middle), and estimated total scores (right) between risk subgroups (based on the estimate R package). (B) Violin plot comparing tumor‐infiltrating immune cell scores between low‐ and high‐risk groups. Kaplan–Meier survival curves of CD8 T cell (C), memory B cell (D), γδ T cell (E), and CD4 memory T cell (F) groups in the entire cohort (divided using their respective optimal infiltration scores as the cutoff value).

An analysis of differences in the TME between risk subgroups divided based on the model established with *FAM182B* and *AGAP11* genes revealed that the low‐risk group had higher scores (Figure 10A, Data S5). An analysis of tumor‐infiltrating immune cells showed statistically significant differences between the low‐ and high‐risk groups in nine groups of immune cells, including CD8 and CD4 memory T cells (Figure 10B, Data S6). Subsequent survival analysis revealed that CD8 T cell (Figure 10C), memory B cell (Figure 10D), γδ T cell (Figure 10E), and CD4 memory T cell (Figure 10F) scores had a significant impact on survival. Furthermore, survival analysis showed that the tumor‐infiltrating immune cell types with poorer prognosis were more prevalent in the high‐risk group.

### 3.9 Reclustering analysis based on the correlation of model lncRNAs

**FIGURE 11:**
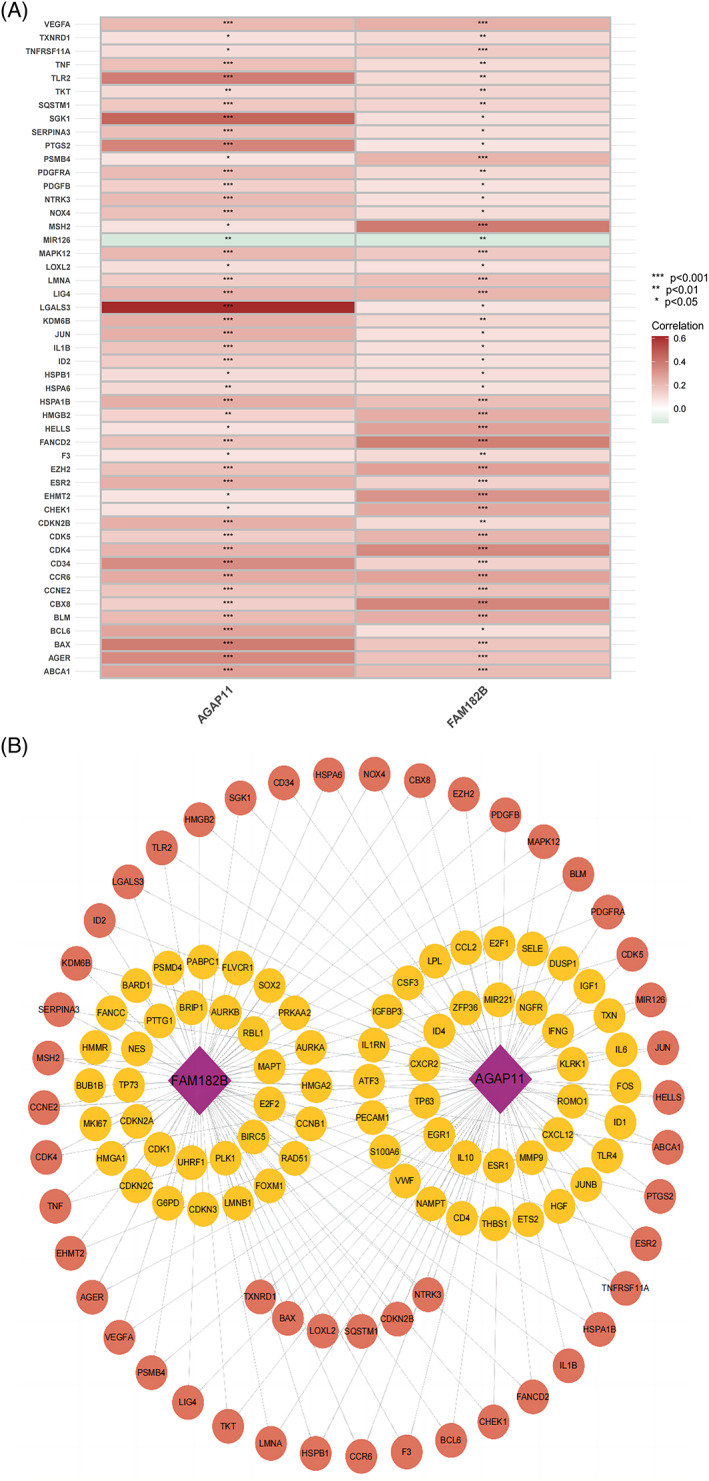
Cellular senescence genes associated with model lncRNAs. (A) Heatmap of correlation between model lncRNAs and cellular senescence genes. (B) Network diagram of model genes and differentially expressed cellular senescence genes. Purple rhombi represent model lncRNAs; circles represent cellular senescence genes, with the color representing the number of related model lncRNAs (two in red and one in yellow).

**FIGURE 12:**
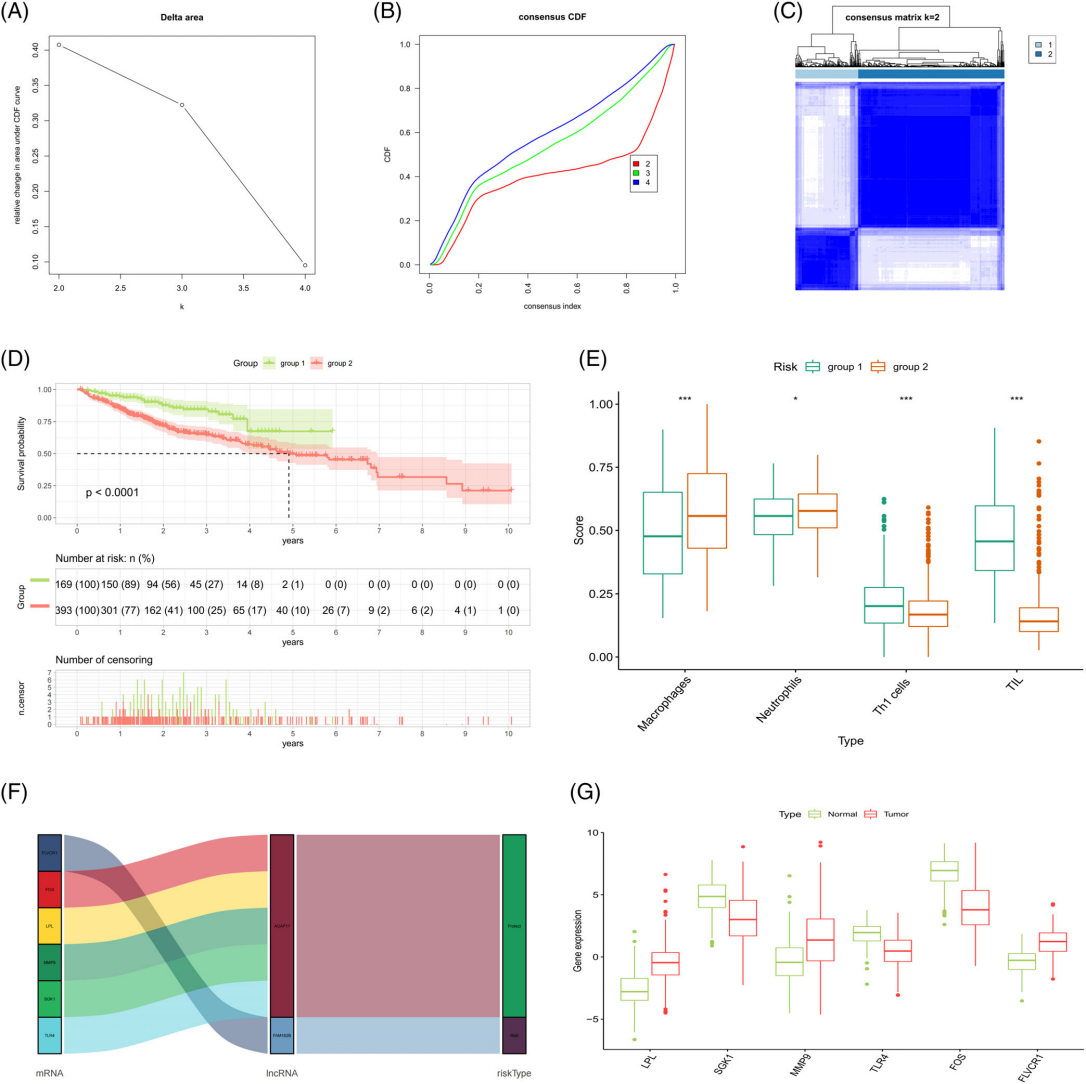
Recluster analysis. (A) Relative change in area under the curve of the cumulative distribution function based on the number of the consensus matrix (*k*). (B) Cumulative distribution function for measuring the distribution of values in a consensus matrix. (C) Heatmap generated based on the consensus matrix with *k* = 2. (D) Kaplan–Meier survival curves for OS in patients from groups 1 and 2. (E) Boxplots comparing differences in immune composition between groups 1 and 2. (F) Sankey diagram of cellular senescence genes significantly correlate with model lncRNAs. (G) Boxplots of cellular senescence genes between tumor and normal tissues.

The correlation heatmap shows the differentially expressed genes associated with model lncRNAs (Figure 11A; Data S7), and the network diagram shows the association between the model lncRNAs and the differentially expressed genes (Figure 11B). Samples consisting of these differentially expressed genes were reclustered and divided into two groups (as the optimal choice) (Figure 12A–C); the survival curves indicated that this grouping had an effect on survival time (Figure 12B). An analysis of immune composition between the recluster‐groups showed that four groups of immune cells, including macrophages and tumor‐infiltrating lymphocytes, exhibited statistically significant differences (Figure 12C). Finally, a Sankey diagram (Figure 12D) and boxplot (Figure 12E) were constructed to visualize the differentially expressed genes closely related to the model lncRNAs ($\left|\text{PCCs}>0.4\right|$, *p* < .001).

### 3.10 Immune escape and drug correlation analysis

**FIGURE 13:**
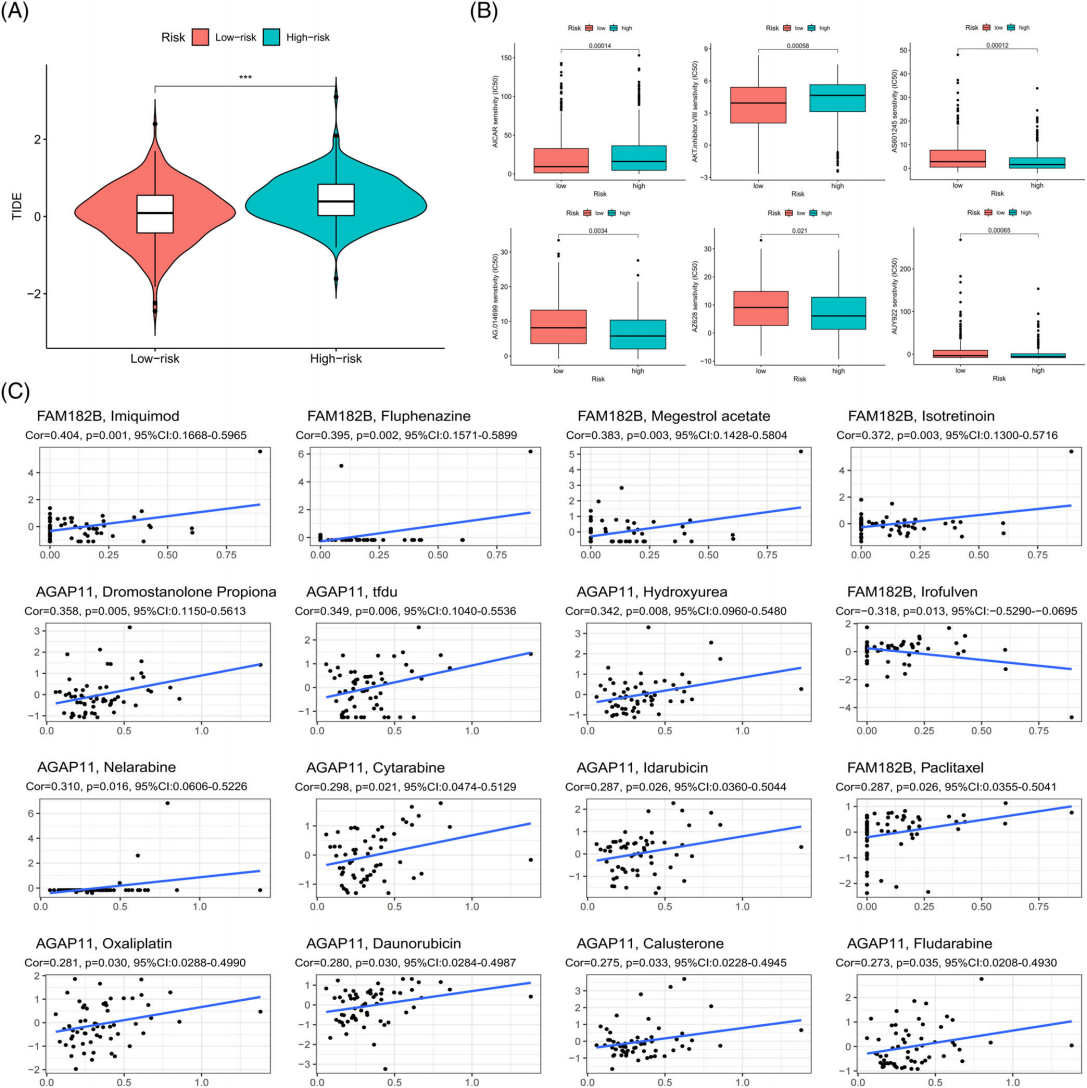
Immune‐targeted drug prediction of model genes. (A) Comparison of TIDE prediction scores between the low‐ and high‐risk groups. (B) Boxplots of therapeutic drugs with different half‐maximal inhibitory concentrations in the risk subgroups. (C) Scatter plot of model lncRNAs and immune‐targeted drugs.

There were significant differences in the immune escape scores between the low‐ and the high‐risk groups, and a higher immune escape score in the high‐risk group indicated a higher possibility of immune escape and a worse prognosis, which was consistent with the prediction results of the model (Figure 13A). Then the difference between the groups in terms of half‐maximal inhibitory concentrations of the drug was used to screen for potentially beneficial therapeutic drugs (Figure 13B); potential drugs predicted from the expression of model lncRNAs had a *p* value of less than .05, which demonstrated the potential of model lncRNAs as therapeutic targets to some extent (Figure 13C).

## 4 DISCUSSION

In this study, the differentially expressed lncRNAs related to cellular senescence were screened using Cox and LASSO regression analyses, which were performed using the DESeq2 and limma R packages. A risk signature containing two lncRNAs, namely, *FAM182B* and *AGAP11* was also established. In this context, the *AGAP11* gene is considered to be protective, and high expression of *AGAP11* improves patient prognosis; however, the *FAM182B* gene confers the opposite effects. Based on subsequent internal validation, Kaplan–Meier survival analysis was performed in the subgroups; the results confirmed the high‐risk group had a significantly shorter survival time than the low‐risk group. On internal validation, the AUC of the temporal ROC curve for each cohort was greater than 0.66; the risk and nomogram scores of the clinical characteristic ROC curves of the entire cohort were also reliable. Additionally, as confirmed by Cox regression analysis, the risk score independently predicted the prognosis in patients with liver cancer. Furthermore, KEGG and GO analyses of genes demonstrated enrichment in tumor invasion and immune‐related signaling pathways, which suggested that patient prognosis may be related to tumor‐infiltrating immune cells and the TME.

TMB was included in the mutation analysis for further investigation of the impact of mutations on the prognosis of risk subgroups; the findings showed that the TMB correlated with risk scores; and that the difference between the subgroups was statistically significant. The H‐TMB and L‐TMB groups exhibited differences in patient survival times. Further combined analysis of TMB and risk subgroups revealed that the combination of H‐TMB + high‐risk conferred the worst prognosis, confirming the validity and accuracy of the prognostic model. The aging process may be influenced by a variety of factors, including immune factors that mediate external signals affecting the aging mechanism. The TME showed that the scores of genes were higher in the low‐risk group compared with those in the high‐risk group, which indicated that the former had a stronger anti‐tumor immune response and better expected treatment response compared with the latter. Among the tumor‐infiltrating immune cells, nine types of immune cells, including CD8 and γδ T cells, differed among the risk subgroups. Among these cell types, memory B, CD8 T, resting CD4 memory T, and γδ T cells influenced patient survival; an increase in the number of these cell types suggested that these patients had a better prognosis. This finding concurs with the generally accepted notion that increased numbers of CD8 T and resting CD4 memory T cells are indicative of better prognosis in cancer.^16^, ^17^ As immune T cells that kill both cancer and tumor stem cells and recognize cancer antigens, γδ T cells are known to inhibit tumor growth by mediating the expression of Ia, LFA‐1, CD56, and CD161 and promoting the production of IFN‐γ and perforin.^18^ Studies have shown that an increase in B cell counts can inhibit tumorigenesis in patients with liver cancer.^19^, ^20^, ^21^ After reclustering, patients in group 1 had a better prognosis compared with the patients in group 2; comparison of immune composition between the two groups showed differences in four types of immune cells, namely, neutrophils, macrophages, Th‐1 cells, and tumor‐infiltrating lymphocytes. Evidence suggests that Th1 lymphocytes support cellular immune responses associated with the differentiation of CD8 T cells into cytotoxic T lymphocytes and also support their survival; this finding is consistent with the high CD8 T cell infiltration observed among the samples in this study.^22^, ^23^ This indicates that the model genes may be associated with tumor‐infiltrating immune cells and the TME, thereby influencing patient prognosis.

We constructed a prognostic model composed of two lncRNAs, namely, *FAM182B* and *AGAP11*; among them, *AGAP11* was considered to be beneficial for patient prognosis in this study. *AGAP11* belongs to the ankyrin repeat and GTPase domain Arf GTPase activating protein gene family. Although no experimental data pertaining to AGAP11 have been reported in the field of cancer, there is evidence to suggest that closely related genes, namely, *LPL*, *MMP9*, *SGK1*, *TLR4*, and *FOS* play certain roles in liver cancer through different mechanisms.^24^, ^25^, ^26^, ^27^, ^28^ Similarly, there are no reports on *FAM182B*, a risk gene included in our model, in the field of cancer. However, there is evidence indicating that *FLVCR1‐AS1*, which was significantly associated with *FAM182B* expression in this study, promotes HCC progression by directly sponging miR‐513c to increase *MET* expression.^29^

In summary, we constructed a cellular senescence‐related lncRNA model to guide the prognosis of liver cancer, and explored its accuracy and efficacy. Although various methods were utilized to optimize our model, the model does have some limitations. Firstly, internal validation was performed on the entire cohort, but external validation was not performed. However, on subsequent analysis of the model via a third‐party website, the low‐ and high‐risk groups showed statistically significant differences; this may, to a certain extent, be regarded as external validation of the model. Secondly, relevant experimental data demonstrating the relationship between the *AGAP11* and *FAM182B* genes and the prognosis of HCC are lacking; however, the significantly related genes used in this study have been confirmed to be involved in the occurrence of liver cancer. We therefore believe that our prognostic model is relatively reliable. Finally, it is expected that the prognostic model can be validated in practice and be helpful in the clinic.

## 5 CONCLUSIONS

Based on database analysis and research, cellular senescence‐related lncRNAs can be used to predict the prognosis of liver cancer patients. This study provides a basis for using cellular senescence‐related lncRNAs as potential prognostic markers in patients with liver cancer.

## AUTHOR CONTRIBUTIONS

**Hao Huang:** Conceptualization (equal); data curation (lead); formal analysis (equal); investigation (equal); methodology (lead); validation (equal); visualization (lead); writing – original draft (equal); writing – review and editing (equal). **Hao Yao:** Investigation (equal); writing – original draft (equal). **Yaqing Wei:** Investigation (equal); validation (equal). **Ming Chen:** Conceptualization (equal); funding acquisition (equal); investigation (equal); project administration (equal); supervision (equal). **Jinjin Sun:** Conceptualization (lead); formal analysis (equal); funding acquisition (lead); project administration (lead); supervision (lead); writing – review and editing (equal).

## CONFLICT OF INTEREST STATEMENT

The authors have stated explicitly that there are no conflicts of interest in connection with this article.

## ETHICS STATEMENT

Since the UCSC Xena, TCGA and ICGC databases are all public and open databases, and the patients involved in the databases have obtained ethical approval, relevant data for research can be obtained for free and related articles can be published without ethical issues and other issues conflict of interest or profit. The study complies with the Declaration of Helsinki and was approved by the Ethics Committee of The Second Hospital of Tianjin Medical University.

## Supporting information


**Data S1**. Supporting Information.

## Data Availability

The data used to support the results are available at the GENCARD (https://www.genecards.org/), UCSCXena (https://xenabrowser.net/datapages/), TCGA (https://portal.gdc.cancer.gov/), ICGC (https://dcc.icgc.org/), GSEA (http://www.gsea-msigdb.org/gsea/index.jsp), TIDE (http://tide.dfci.harvard.edu/), and CellMiner (https://discover.nci.nih.gov/cellminer/home.do) databases.
