# Supplementary material for: Cellular senescence‐related long noncoding ribonucleic acids: Predicting prognosis in hepatocellular carcinoma
Source: Cancer Rep (Hoboken). 2023 Feb 1;6(4):e1791. doi: 10.1002/cnr2.1791 (PMC10075286; doi:10.1002/cnr2.1791)
Supplement: Supplementary file 1 — Data S1. Supporting Information. [file CNR2-6-e1791-s001.pdf]

Risk score and risk grouping for the training cohort.

Edited ID	OS(year)	status	AGAP11	FAM182B	Riskscore	Riskgroup
S06Q-DO-002T	0.246575342	1	0.184012109	0	0.737463645	low
S07Q-DO-003T	1.561643836	1	0.055179616	0	1.139152751	low
S10Q-DO-006T	2.054794521	0	0.237955236	0.130345083	0.712780421	low
S14Q-DO-010T	1.643835616	0	0.463804168	0.816175826	0.724688127	low
S15Q-DO-011T	0.575342466	0	0.168004747	0.035678196	0.810587768	low
S17Q-DO-013T	0.410958904	0	0.623837933	0.014103349	0.169825934	low
S18Q-DO-014T	3.369863014	0	0.335478797	0.382417588	0.68285025	low
S21Q-DO-017T	1.643835616	0	0.105339903	0.175170327	1.173408316	low
S23Q-DO-019T	1.97260274	0	0.407469359	1.462678485	1.82630156	high
S24Q-DO-020T	2.630136986	0	0.415628234	0.180175365	0.41409513	low
S25Q-DO-021T	1.890410959	0	0.541069523	0.523171698	0.400304723	low
S26Q-DO-022T	3.698630137	0	0.141485632	0.504294141	1.509338087	high
S30Q-DO-026T	0.904109589	0	0.401245769	0.745974478	0.826468399	low
S32Q-DO-028T	1.479452055	0	0.225430051	0.018884025	0.655154858	low
S33Q-DO-029T	2.383561644	0	0.060849783	1.5120883	6.22317715	high
S34Q-DO-030T	2.219178082	0	0.561780688	0.486114005	0.357895425	low
S36Q-DO-032T	1.150684932	1	0.310595595	0.978860508	1.462031016	high
S38Q-DO-034T	2.219178082	1	0.699246005	0.077745926	0.141533514	low
S39Q-DO-035T	3.123287671	0	0.254460046	0.1685346	0.704040647	low
S43Q-DO-039T	2.876712329	0	0.51907294	0.019890111	0.243456523	low
S46Q-DO-042T	1.479452055	0	0.282138839	1.504362591	2.92306665	high
S49Q-DO-045T	4.273972603	0	0.19999654	0.135107343	0.814599075	low
S50Q-DO-046T	1.97260274	1	0.450741775	0.317886934	0.430079294	low
S54Q-DO-050T	1.068493151	0	0.335514355	0.620983742	0.895222415	low
S58Q-DO-054T	0.575342466	0	0.588197241	0.283223422	0.259999281	low
S60Q-DO-056T	5.424657534	0	0.665087917	0.050593581	0.154005664	low
S64Q-DO-060T	1.726027397	0	0.496794466	0.069258155	0.277603301	low
S65Q-DO-061T	0.328767123	1	0.129799657	0.197548265	1.1082374	low
S67Q-DO-063T	5.917808219	0	0.161128319	1.113633163	2.821709084	high
S68Q-DO-064T	4.356164384	0	0.449666592	0.47188915	0.514134012	low
S69Q-DO-065T	1.561643836	0	0.734542112	0.127674889	0.132967929	low
S70Q-DO-066T	3.287671233	0	0.247876001	0.879040091	1.61309612	high
S72Q-DO-068T	1.808219178	0	0.283689026	0.434884494	0.86320581	low
S73Q-DO-069T	2.876712329	0	0.865202395	0.912536437	0.208597686	low
S75Q-DO-071T	1.561643836	1	0.148836902	0.300942416	1.168747776	low
S76Q-DO-072T	0.246575342	0	0.166876864	0.381823244	1.205500337	low
S77Q-DO-073T	1.068493151	0	0.056927515	0.394191983	1.771862676	high
S83Q-DO-079T	1.397260274	0	0.171610111	0.256565789	1.029091783	low
S85Q-DO-081T	2.547945205	0	0.542811839	0.366273193	0.333009502	low
S86Q-DO-082T	4.191780822	0	0.535709	0.187138473	0.278305043	low
S90Q-DO-086T	2.054794521	0	0.410222449	1.074462963	1.164325475	low
S93Q-DO-089T	1.561643836	0	0.156138086	0.124653697	0.933416746	low
S97Q-DO-093T	3.945205479	1	0.21624401	0.062603746	0.710183774	low
S98Q-DO-094T	2.465753425	1	0.131845222	0.180982676	1.08010135	low
S02W-DO-097T	3.945205479	1	0.37853404	0	0.382483198	low
S03W-DO-098T	2.301369863	0	0.333431978	0.144649434	0.524881958	low
S06W-DO-101T	2.547945205	0	0.191281159	0.194956569	0.897915889	low

For Review Only

S08W-DO-103T	2.383561644	0	0.309535594	0.053779911	0.513183993	low
S09W-DO-104T	3.534246575	0	0.254023261	0.01394755	0.59155854	low
S10W-DO-105T	3.698630137	0	0.3419763	0.133075518	0.503302823	low
S15W-DO-110T	1.397260274	1	0.04018306	0.292820731	1.671009738	high
S16W-DO-111T	4.356164384	0	0.900165915	0.113528752	0.074817044	low
S24W-DO-119T	2.219178082	0	0.389111135	0.725392687	0.84112863	low
S28W-DO-123T	0.575342466	0	0.074584241	0.296389625	1.493874838	high
S29W-DO-124T	2.301369863	0	0.310072499	0.209164111	0.611110147	low
S30W-DO-125T	1.561643836	0	0.357037215	0.297375506	0.576479357	low
S31W-DO-126T	1.643835616	0	0.554143917	0.60379149	0.419746409	low
S33W-DO-128T	0.410958904	1	0.343901921	0.101027876	0.48217129	low
S35W-DO-130T	1.315068493	1	0.358356424	1.128176582	1.474313754	high
S37W-DO-132T	3.534246575	0	0.5907097	0.252703757	0.24902189	low
S41W-DO-136T	3.863013699	0	0.694245134	0.028256662	0.136076171	low
S43W-DO-138T	2.876712329	0	0.999918558	0.01933147	0.048009515	low
S46W-DO-141T	1.397260274	0	0.15097215	0.10898741	0.933085334	low
S47W-DO-142T	3.287671233	0	0.547269962	1.081518249	0.739045421	low
S49W-DO-144T	2.465753425	0	0.796610462	0.232070666	0.121409966	low
S51W-DO-146T	3.452054795	0	1.539992045	0	0.007588556	low
S52W-DO-147T	1.643835616	0	0.27687919	0.150852735	0.639757707	low
S60W-DO-155T	1.068493151	0	0.087688305	0.642844887	2.118231654	high
S63W-DO-158T	1.97260274	0	0.09271788	0.007961026	1.012709843	low
S67W-DO-162T	3.452054795	0	0.537860807	0.330513426	0.325145364	low
S69W-DO-164T	1.643835616	0	0.252629407	0.027788967	0.603764015	low
S78W-DO-173T	0.657534247	0	1.156902668	0.227408639	0.035796656	low
S79W-DO-174T	0.904109589	0	0.095810539	0.015252613	1.010527079	low
S80W-DO-175T	3.616438356	0	0.31313496	0.648756622	0.996395239	low
S81W-DO-176T	1.726027397	0	0.12662933	0.008051954	0.903280886	low
S83W-DO-178T	2.547945205	0	0.402386346	0.024508863	0.36285853	low
S84W-DO-179T	1.232876712	0	0.264390983	0.076787487	0.613469017	low
S85W-DO-180T	1.315068493	0	0.283400412	0.231407585	0.685780839	low
S91W-DO-186T	0.821917808	0	0.115647184	0.064200983	0.999107636	low
S92W-DO-187T	0.739726027	1	0.081536941	1.462207315	5.483887201	high
S93W-DO-188T	3.452054795	1	0.328739516	1.29845128	1.976875202	high
S94W-DO-189T	1.561643836	0	0.282453921	0.09487073	0.589161794	low
S96W-DO-191T	0.082191781	1	0.441299147	0.099073662	0.346316912	low
S99W-DO-194T	3.698630137	0	0.051624588	0.265062731	1.557823451	high
S01R-DO-195T	4.602739726	0	0.876733342	0.19730896	0.08905679	low
S02R-DO-196T	3.205479452	0	0.520126312	0.080749406	0.259951528	low
S03R-DO-197T	2.301369863	0	0.39551342	0.09881031	0.404069497	low
S05R-DO-199T	0.821917808	0	0.428539279	1.269089416	1.365252279	low
S13R-DO-207T	1.068493151	0	0.132879484	0	0.876375632	low
S15R-DO-209T	3.123287671	0	0.126179552	0.098166006	1.00213246	low
S16R-DO-210T	1.97260274	0	0.182350078	0.175156783	0.904819096	low
S17R-DO-211T	2.136986301	0	0.411758895	0.62514001	0.695380163	low
S19R-DO-213T	2.95890411	0	3.483228908	0.077636302	1.18E-05	low
S20R-DO-214T	0.493150685	1	0.063419968	0.561529578	2.09624541	high
S21R-DO-215T	2.465753425	0	0.321972183	0.167479196	0.559910516	low
S24R-DO-218T	3.780821918	0	0.630158664	0.37285896	0.24984609	low

For Review Only

S25R-DO-219T	2.630136986	0	0.20744311	0.127430892	0.787486077	low
S28R-DO-222T	1.232876712	0	0.298498437	0.043695889	0.526596477	low
S31R-DO-225T	1.068493151	0	0.496696107	0.10991759	0.290818133	low
S32R-DO-226T	2.465753425	0	1.128634565	0.358458942	0.045699326	low
S34R-DO-228T	4.356164384	0	0.388443826	0.15122797	0.439208045	low
S35R-DO-229T	2.547945205	0	0.165096662	0.301965566	1.107623118	low
S36R-DO-230T	1.397260274	0	0.138681986	0.615504156	1.728794449	high
S38R-DO-232T	1.97260274	0	0.437082077	0.033429105	0.326046867	low
S40R-DO-234T	2.383561644	0	0.28396052	0.24059533	0.691664709	low
S41R-DO-235T	0.904109589	0	0.231187596	0.491321676	1.098764114	low
S42R-DO-236T	2.876712329	0	0.448439849	0.53898456	0.557140885	low
S43R-DO-237T	3.205479452	0	0.621321411	0.012116746	0.170888539	low
S45R-DO-239T	0.328767123	1	0.307234287	3.009884589	14.84421132	high
TCGA-DD-AAVS	4.994520548	0	0.008238454	0.083326723	1.467180173	high
TCGA-5R-AA1D	1.230136986	0	0.038321	0.030686507	1.248611469	low
TCGA-XR-A8TC	3.668493151	0	0.033397501	0.052983219	1.302088706	low
TCGA-2Y-A9GS	1.983561644	1	0.01853133	0.252183831	1.716620505	high
TCGA-CC-A1HT	0.276712329	1	0.005703347	0.281098202	1.852412058	high
TCGA-DD-AACL	0.293150685	1	0	0.317517794	1.968154356	high
TCGA-G3-AAUZ	1.315068493	0	0	0.033302584	1.425240199	high
TCGA-2Y-A9H1	3.367123288	1	0	0	1.372346186	low
TCGA-DD-AACI	4.432876712	0	0	0.014672794	1.395404448	high
TCGA-EP-A26S	1.665753425	0	0	0.032681684	1.424235622	high
TCGA-LG-A6GG	1.060273973	0	0	0.025941508	1.413375896	high
TCGA-4R-AA8I	0.717808219	1	0.011024831	0.280277791	1.817744361	high
TCGA-DD-AADK	2.873972603	0	0.016360645	0.013081322	1.318056253	low
TCGA-EP-A3RK	0.994520548	0	0	0.092898156	1.525034934	high
TCGA-ZP-A9CV	2.980821918	1	0	0.039962187	1.436059681	high
TCGA-ES-A2HS	1.884931507	1	0	0.00731758	1.383797721	low
TCGA-2Y-A9H8	1.734246575	1	0	0.022906309	1.408512681	high
TCGA-G3-A5SK	2.038356164	0	0.010551008	0.025151847	1.362707575	low
TCGA-DD-AAW3	4.473972603	0	0	0.042725448	1.440573071	high
TCGA-ED-A4XI	2.243835616	0	0.034305656	0.06107365	1.310085074	low
TCGA-CC-5263	0.353424658	1	0	0.099777916	1.536996211	high
TCGA-DD-A3A9	2.550684932	1	0	0.047988548	1.449208865	high
TCGA-G3-A5SM	1.424657534	0	0	0.072687853	1.490432611	high
TCGA-DD-A11A	0.216438356	0	0	0.070401405	1.486567725	high
TCGA-ZP-A9D0	2.989041096	0	0	0.006855713	1.383072113	low
TCGA-DD-A4NN	2.463013699	1	0	0.019584146	1.403208851	high
TCGA-UB-A7MC	1.369863014	0	0.111378653	0.021596688	0.965733253	low
TCGA-DD-A1EA	6.616438356	0	0	0.03184383	1.422881149	high
TCGA-DD-A4NG	2.197260274	1	0	0.044184272	1.442961568	high
TCGA-08-A75V	1.473972603	0	0	0.024411118	1.410921699	high
TCGA-DD-A39V	1.761643836	1	0	0	1.372346186	low
TCGA-BC-A69I	1.060273973	0	0	0.007604607	1.384248843	low
TCGA-ZS-A9CF	6.608219178	0	0.00999568	0.068516656	1.434179799	high
TCGA-DD-A4ND	7.523287671	0	0.026890661	0.070507487	1.357753596	low
TCGA-BC-A217	3.82739726	1	0	0.848735618	3.597895895	high
TCGA-G3-A25S	1.139726027	1	0	0.03048426	1.420686011	high

For Review Only

TCGA-NI-A4U2	4.906849315	1	0.004013351	0.038011557	1.413603903	high
TCGA-DD-AAW2	5.082191781	0	0.029648252	0	1.241668248	low
TCGA-DD-A4NH	2.512328767	0	0.009826222	0.038844855	1.387452935	high
TCGA-DD-AAEB	1.309589041	0	0	0.022481321	1.407833071	high
TCGA-BD-A3EP	1.120547945	0	0.022069832	0.221820053	1.638749573	high
TCGA-CC-A7IK	0.717808219	1	0.067532658	0.395894276	1.712871159	high
TCGA-RC-A7SH	1.282191781	0	0.023311753	0.055236505	1.350627062	low
TCGA-ED-A7XO	1.169863014	0	0	0.167916527	1.660648956	high
TCGA-DD-AAW1	5.449315068	0	0.007980255	0.050243623	1.414313798	high
TCGA-G3-A7M7	0.989041096	0	0	0.007973787	1.384829299	low
TCGA-CC-5261	0.265753425	1	0	0.341363803	2.022179413	high
TCGA-WQ-A9G7	0.082191781	0	0	0.043912469	1.442516252	high
TCGA-WX-AA47	1.523287671	1	0	0.017331985	1.399624642	high
TCGA-ZP-A9CZ	1.934246575	0	0.010040435	0.016004436	1.35095127	low
TCGA-DD-AA3A	1.123287671	1	0	0.132520779	1.595222011	high
TCGA-DD-A39Y	0.468493151	1	0	0.034464221	1.427121558	high
TCGA-DD-A4NI	2.235616438	0	0.00690338	0.005516044	1.349165461	low
TCGA-UB-A7MA	2.323287671	0	0.042297909	0.042227604	1.248216505	low
TCGA-CC-A3MB	0.863013699	1	0	0.059576026	1.468404711	high
TCGA-KR-A7K2	2.271232877	0	0	0.01823891	1.401066868	high
TCGA-RC-A7SB	1.610958904	0	0	0.230876237	1.783728344	high
TCGA-GJ-A9DB	0.183561644	1	0.010009264	0.222565452	1.708277694	high
TCGA-DD-AAVY	5.397260274	0	0.017083819	0	1.295455185	low
TCGA-BC-A10W	0.249315068	1	0.007167052	0.141973014	1.573901162	high
TCGA-5R-AA1C	1.424657534	0	0	0.016858481	1.398872249	high
TCGA-5R-AAAM	0.126027397	1	0	0.033466024	1.425504752	high
TCGA-DD-A3A1	0.638356164	1	0	0.015134966	1.396137011	high
TCGA-FV-A3R2	0.531506849	1	0.008196106	0	1.334903622	low
TCGA-DD-AACN	3.567123288	0	0.021761686	0.051601286	1.352118261	low
TCGA-DD-A1EJ	2.753424658	1	0	0.20198408	1.726153816	high
TCGA-FV-A2QQ	1.997260274	0	0	0.096664955	1.531572383	high
TCGA-G3-AAV7	0.989041096	0	0.030147954	0.109298049	1.403386954	high
TCGA-DD-A11C	1.81369863	0	0.013299122	0.112844482	1.491500258	high
TCGA-NI-A8LF	2.189041096	0	0.093263048	0.099109544	1.121086557	low
TCGA-DD-A73G	9.528767123	0	0	0.014752051	1.395530047	high
TCGA-DD-AAVZ	5.205479452	0	0.010418139	0.081178345	1.452877302	high
TCGA-DD-AACF	1	1	0	0	1.372346186	low
TCGA-MI-A75C	0.797260274	0	0	0.014666696	1.395394785	high
TCGA-DD-AAVX	4.706849315	0	0	0.024507756	1.411076546	high
TCGA-ZS-A9CE	3.4	0	0.013606637	0.14034467	1.537217433	high
TCGA-CC-5264	0.279452055	1	0	0.747153542	3.205897911	high
TCGA-G3-A5SJ	1.912328767	0	0.009779879	0.068865106	1.435792801	high
TCGA-2Y-A9H6	0.978082192	0	0	0.056831817	1.463835794	high
TCGA-CC-A8HT	0.383561644	1	0	0.107148837	1.549915548	high
TCGA-WQ-AB4B	1.082191781	0	0	0.011275174	1.390030863	high
TCGA-DD-A11D	4.273972603	1	0	0	1.372346186	low
TCGA-K7-A5RF	1.728767123	0	0.009215164	0.007364425	1.341492417	low
TCGA-DD-A73B	0.775342466	1	0.00793379	0.031424177	1.384625706	low
TCGA-PD-A5DF	1.750684932	1	0	0.133463967	1.596931549	high

For Review Only

TCGA-ZS-A9CD	3.797260274	1	0.022015586	0.069184724	1.37820693	low
TCGA-DD-A1ED	6.304109589	0	0.019842342	0.010598692	1.298990904	low
TCGA-DD-AAEK	2.923287671	0	0.065037871	0.013222012	1.118543176	low
TCGA-K7-A6G5	1.402739726	0	0.008711356	0	1.33258421	low
TCGA-WX-AA46	2.071232877	0	0	0	1.372346186	low
TCGA-DD-AACP	1.136986301	0	0	0.165365428	1.655844956	high
TCGA-DD-AAD6	1.84109589	0	0	0.042915553	1.440884101	high
TCGA-K7-A5RG	1.421917808	0	0	0.194629852	1.711797892	high
TCGA-G3-AAV5	0.969863014	0	0	0.012204702	1.39149892	high
TCGA-2Y-A9H4	3.978082192	0	0	0.160738356	1.647167104	high
TCGA-DD-A39Z	1.646575342	1	0.011051733	0.017612789	1.348809301	low
TCGA-CC-5260	0.238356164	1	0	0.51985728	2.476567963	high
TCGA-DD-A116	4.443835616	1	0.018192354	0	1.290617393	low
TCGA-BC-A3KG	1.863013699	0	0	0.295097623	1.918676831	high
TCGA-DD-A1EL	1.136986301	1	0.017696773	0.082895644	1.420387823	high
TCGA-G3-A25V	2.356164384	0	0	0.033753866	1.425970791	high
TCGA-DD-AADB	3.402739726	0	0	0.007596978	1.384236851	low
TCGA-GJ-A3OU	2.408219178	0	0	0.04970226	1.452031912	high
TCGA-BD-A2L6	3.734246575	0	0	0.017043142	1.399165625	high
TCGA-2Y-A9GX	6.690410959	0	0	0.007932758	1.384764779	low
TCGA-MI-A75G	1.912328767	0	0	0	1.372346186	low
TCGA-CC-A8HS	0.821917808	1	0.152173163	0.122796782	0.943998984	low
TCGA-DD-AACY	3.97260274	0	0	0.0953351	1.529261165	high
TCGA-CC-A7IE	0.594520548	1	0	0.108863719	1.552936836	high
TCGA-2Y-A9HA	0.098630137	1	0.007200613	0.126854278	1.546934434	high
TCGA-G3-A6UC	1.838356164	0	0.008927959	0.035326093	1.386115819	high
TCGA-CC-A7IH	1	0	0.008166783	0.070213295	1.445843009	high
TCGA-MI-A75E	1.389041096	0	0	0.061754579	1.472042002	high
TCGA-DD-AAD5	3.684931507	0	0	0.133397981	1.59681189	high
TCGA-DD-A39W	2.265753425	1	0	0.040403521	1.436779588	high
TCGA-2Y-A9GT	4.449315068	1	0	0.026754346	1.41468113	high
TCGA-DD-A39X	4.64109589	1	0	0.012897646	1.392594335	high
TCGA-2Y-A9GV	6.936986301	1	0.148169062	0.020622825	0.852020187	low
TCGA-DD-AACC	4.616438356	1	0.012200132	0.029060221	1.361171851	low
TCGA-MR-A8JO	0.904109589	0	0.116867709	0.078824461	1.011661787	low
TCGA-G3-A25U	4.482191781	0	0.008443665	0.013463605	1.354338143	low
TCGA-BC-A10R	0.843835616	1	0.014107022	0.06638027	1.410988913	high
TCGA-DD-AACS	4.942465753	0	0.013941659	0.022205213	1.342701425	low
TCGA-CC-A7IL	0.761643836	1	0.020695697	0.022027989	1.312175862	low
TCGA-CC-A7IJ	1.046575342	0	0	0.020079876	1.403999014	high
TCGA-DD-AADA	3.378082192	0	0.009231285	0.043708416	1.397941365	high
TCGA-FV-A4ZP	6.810958904	1	0	0.027215722	1.415422534	high
TCGA-G3-A7M9	0.153424658	1	0.024943067	0.964771005	3.77323959	high
TCGA-DD-A4NE	1.808219178	1	0.018092229	0.070929262	1.399347667	high
TCGA-DD-A119	0.610958904	1	0	0.05372758	1.458684589	high
TCGA-G3-A3CH	2.136986301	0	0	0.085423133	1.512144215	high
TCGA-DD-AACZ	0.468493151	1	0	0.118864813	1.570674511	high
TCGA-WJ-A86L	0.945205479	0	0	0.340696705	2.020648074	high
TCGA-LG-A9QC	1.164383562	0	0	0.034279029	1.42682146	high

For Review Only

TCGA-ED-A82E	1.117808219	0	0.024223823	0.142318148	1.486435375	high
TCGA-DD-AAD3	3.547945205	0	0	0.055202972	1.461130606	high
TCGA-KR-A7K8	2.482191781	0	0.011029325	0.126887785	1.527131182	high
TCGA-CC-A3MC	0.994520548	0	0.008979961	0.042532167	1.397260021	high
TCGA-UB-A7MB	1.646575342	0	0	0.055585351	1.461765213	high
TCGA-BC-A216	3.701369863	0	0.016402781	0.491985331	2.270184503	high
TCGA-DD-AAVV	6.726027397	0	0.010551157	0.041679987	1.388525649	high
TCGA-DD-AADR	5.556164384	0	0	0.009385034	1.387050431	high
TCGA-CC-A8HV	0.764383562	1	0	0.072299318	1.489775145	high
TCGA-DD-AAEI	4.194520548	0	0.009173924	0.057634149	1.420499281	high
TCGA-CC-A3MA	0.830136986	1	0.008831775	0.19802047	1.667935178	high
TCGA-FV-A3I1	0.676712329	1	0.017598452	0.204051547	1.630435076	high
TCGA-CC-A123	0.6	0	0	0.008381509	1.385470639	low
TCGA-CC-A7II	1.093150685	0	0.008685532	0.132844274	1.549706707	high
TCGA-UB-A7MF	0.58630137	1	0.006912427	0.059623287	1.434620105	high
TCGA-DD-A1EC	1.649315068	0	0	0.011103383	1.389759713	high
TCGA-DD-AACE	5.983561644	0	0.012471155	0	1.315780934	low
TCGA-ED-A66X	1.112328767	0	0.016626149	0.193439207	1.616199015	high
TCGA-DD-AACW	3.901369863	0	0.010697485	0.017049473	1.349559341	low
TCGA-DD-AAD0	0.375342466	0	0	0.039329379	1.435028072	high
TCGA-DD-A4NA	2.761643836	0	0	0.092578458	1.524481371	high
TCGA-ED-A459	2.493150685	0	0.008763591	0.18428258	1.642493923	high
TCGA-DD-AAD8	3.339726027	0	0.040665298	0.105820517	1.349106233	low
TCGA-XR-A8TD	2.821917808	0	0.043762861	0.251726242	1.575667221	high
TCGA-2Y-A9H7	3.2	0	0	0.006105286	1.381893978	low
TCGA-RG-A7D4	3.008219178	0	0	0.166314005	1.657629606	high
TCGA-XR-A8TG	2.460273973	0	0.025912502	0.176665787	1.536776012	high
TCGA-DD-AAC9	0.950684932	0	0	0.052886439	1.457291915	high
TCGA-DD-A3A7	1.147945205	1	0	0.074440055	1.493401237	high
TCGA-DD-AAVW	6.347945205	0	0.02162453	0.239907569	1.675273929	high
TCGA-2Y-A9H9	1.909589041	0	0.014798989	0.040992248	1.367691963	low
TCGA-G3-A25X	4.873972603	0	0	0.119446289	1.57171201	high
TCGA-DD-A4NO	6.150684932	0	0.017297802	0.054547893	1.377244418	low
TCGA-DD-AAD1	1.545205479	0	0.056655911	0.206069282	1.432348196	high
TCGA-DD-AADW	1.608219178	0	0.029190777	0.107740676	1.405440226	high
TCGA-DD-A118	9.416438356	0	0	0.090522128	1.520925591	high
TCGA-DD-AACX	0.465753425	0	0	0.052896536	1.457308625	high
TCGA-DD-AADG	3.136986301	0	0.009354816	0.16284976	1.599804014	high
TCGA-G3-A25Z	1.794520548	0	0	0.042772886	1.440650677	high

For Review Only

Risk score and risk grouping for the validation cohort.

Edited ID	OS(year)	status	AGAP11	FAM182B	Riskscore	Riskgroup
S05Q-DO-001T	1.890410959	1	0.143667694	0	0.84503957	low
S08Q-DO-004T	4.02739726	0	0.255863491	0.256065011	0.773943739	low
S09Q-DO-005T	1.97260274	0	0.22248471	0.242964255	0.853443062	low
S12Q-DO-008T	2.794520548	0	0.195726429	1.334706132	3.227222247	high
S13Q-DO-009T	2.219178082	0	0.150767228	0.489204829	1.437936867	high
S16Q-DO-012T	1.479452055	0	0.110019976	0.012211261	0.959886657	low
S19Q-DO-015T	1.561643836	0	0.586358642	0.269247559	0.257498344	low
S20Q-DO-016T	4.02739726	0	0.409579065	0.315747411	0.49297992	low
S27Q-DO-023T	1.561643836	1	0.19926904	0.213619898	0.892753585	low
S28Q-DO-024T	3.945205479	0	0.673094145	0.061556656	0.151777742	low
S29Q-DO-025T	1.315068493	0	0.421396964	0.251161718	0.440203899	low
S31Q-DO-027T	3.287671233	0	0.729365257	0.006935421	0.117974465	low
S35Q-DO-031T	1.479452055	0	0.096724359	0.023950163	1.017414684	low
S37Q-DO-033T	1.890410959	0	0.15194322	0.176614346	1.004270413	low
S41Q-DO-037T	1.808219178	1	0.196737082	0.070818967	0.765623819	low
S42Q-DO-038T	4.602739726	0	0.559840172	0.04798118	0.219037947	low
S45Q-DO-041T	1.397260274	0	0.324768675	0.474701428	0.786207892	low
S47Q-DO-043T	2.219178082	0	0.167173693	0.299042045	1.09624009	low
S48Q-DO-044T	2.383561644	0	0.156055352	0.048386056	0.856214723	low
S51Q-DO-047T	1.643835616	0	0.244695783	0.042558768	0.630638552	low
S52Q-DO-048T	1.890410959	0	0.375748882	0.334970023	0.564803654	low
S53Q-DO-049T	2.630136986	0	0.177473278	0.201386826	0.94764663	low
S55Q-DO-051T	1.315068493	0	0.234995787	0.013763399	0.630662758	low
S56Q-DO-052T	1.890410959	0	0.146786844	0.169209504	1.013343914	low
S57Q-DO-053T	1.97260274	1	0	0.036807208	1.430923765	high
S61Q-DO-057T	2.630136986	0	1.078973878	0.040049188	0.037641467	low
S62Q-DO-058T	2.95890411	0	0.70929143	0.332646301	0.182746143	low
S63Q-DO-059T	2.794520548	0	2.574945074	0.087950329	0.000254983	low
S66Q-DO-062T	1.232876712	0	0.301113147	0	0.496701091	low
S71Q-DO-067T	3.287671233	1	0.278705855	0.552752428	1.00357424	low
S74Q-DO-070T	3.452054795	0	0.140572411	0	0.853913894	low
S78Q-DO-074T	1.97260274	0	0.333765131	0.191589812	0.55299814	low
S80Q-DO-076T	3.04109589	1	0.097399878	0.026385722	1.017909147	low
S81Q-DO-077T	2.465753425	0	0.193355557	0.682113449	1.550437279	high
S82Q-DO-078T	3.287671233	0	0.436267079	0.005628069	0.31678423	low
S84Q-DO-080T	1.97260274	0	0.299604319	0.57169535	0.955562599	low
S87Q-DO-083T	0.246575342	1	0.186805165	0.110090449	0.827831031	low
S88Q-DO-084T	3.452054795	0	0.163173703	0.055414402	0.84258742	low
S89Q-DO-085T	3.287671233	0	1.828031552	0.481402179	0.004958779	low
S91Q-DO-087T	1.479452055	1	0.28177717	0.035382333	0.551934675	low
S92Q-DO-088T	1.726027397	0	0.399232684	2.068794612	3.737437934	high
S94Q-DO-090T	2.136986301	1	0.829589324	0	0.083456496	low
S95Q-DO-091T	1.726027397	0	1.269733372	0.92703733	0.054138195	low
S01W-DO-096T	0.739726027	1	0.3914213	0.243915074	0.48307878	low
S04W-DO-099T	1.643835616	0	0.473317381	0.069855088	0.300698662	low
S05W-DO-100T	0.657534247	1	0.731172639	0.080296829	0.127444068	low
S11W-DO-106T	2.712328767	0	0.522272238	0.272544496	0.320876299	low

For Review Only

S12W-DO-107T	0.657534247	1	0.145202804	0.837516706	2.176099732	high
S13W-DO-108T	3.452054795	0	0.351865065	0.405858428	0.663540373	low
S14W-DO-109T	2.712328767	0	0.860419615	0.149191717	0.089094062	low
S17W-DO-112T	3.616438356	0	0.315159854	0.043884522	0.497907706	low
S18W-DO-113T	3.452054795	0	0.171070623	0	0.77038907	low
S19W-DO-114T	1.397260274	0	0.609349608	0.657902273	0.370470609	low
S21W-DO-116T	4.438356164	0	0.611448898	0.040760854	0.182520164	low
S22W-DO-117T	2.712328767	0	0.331306457	0.132823142	0.521608482	low
S23W-DO-118T	1.561643836	1	0.386919407	0.346358759	0.550986081	low
S25W-DO-120T	1.232876712	0	1.18602127	0.381702109	0.038659494	low
S26W-DO-121T	2.630136986	0	0.083263851	0	1.036135087	low
S27W-DO-122T	0.98630137	1	0.032744036	0.213301364	1.565543543	high
S32W-DO-127T	3.04109589	0	0.3657605	0.186595889	0.49358534	low
S34W-DO-129T	2.136986301	0	0.54495892	0.008151559	0.220134807	low
S40W-DO-135T	1.315068493	1	0.084967395	0.205977605	1.301681428	low
S42W-DO-137T	0.493150685	1	1.773777373	0.284358247	0.004761246	low
S44W-DO-139T	1.068493151	0	0.499405481	0.096458338	0.283799868	low
S45W-DO-140T	1.315068493	0	0.337664857	0.672763855	0.942575213	low
S53W-DO-148T	1.643835616	0	0.168755239	0.98283328	2.370413126	high
S54W-DO-149T	2.876712329	0	0.38584548	0.045815647	0.393089085	low
S55W-DO-150T	2.712328767	0	0.116473966	0	0.926269435	low
S56W-DO-151T	1.479452055	0	0.192441391	0.037585359	0.748034538	low
S57W-DO-152T	0.98630137	0	0.615984694	0.718481662	0.388065516	low
S58W-DO-153T	2.95890411	0	1.144819082	0.015154453	0.029300401	low
S61W-DO-156T	0.657534247	0	0.541887583	0.640564385	0.456129429	low
S62W-DO-157T	2.465753425	0	0.811357337	0.059041824	0.094908047	low
S64W-DO-159T	3.04109589	0	0.134419775	2.140752157	9.9133976	high
S65W-DO-160T	3.205479452	0	0.350824787	1.145399649	1.54214034	high
S68W-DO-163T	2.054794521	0	0.387485628	0.055506182	0.395244754	low
S70W-DO-165T	2.465753425	0	0.283663545	0.060062675	0.564020202	low
S71W-DO-166T	2.712328767	0	0.07047972	0.222451818	1.392725259	high
S72W-DO-167T	3.287671233	0	0.64087539	0.11974296	0.18077181	low
S73W-DO-168T	2.794520548	0	0.297161946	0.139405709	0.58971171	low
S74W-DO-169T	1.150684932	0	0.450915618	0.035864193	0.31203544	low
S75W-DO-170T	3.205479452	0	0.262457573	0.409474033	0.900955013	low
S76W-DO-171T	2.876712329	0	0.298667699	0.28975091	0.695954507	low
S77W-DO-172T	3.534246575	0	0.108934221	0.173127561	1.156573357	low
S82W-DO-177T	2.465753425	0	0.185709521	0.840548071	1.904581198	high
S86W-DO-181T	2.547945205	0	0.231796109	0.244467338	0.828451902	low
S88W-DO-183T	2.547945205	0	0.199177182	0.418117825	1.126475774	low
S90W-DO-185T	1.726027397	0	0.16530149	0.12926211	0.909738915	low
S95W-DO-190T	1.890410959	0	0.147992386	0.372113086	1.270746995	low
S97W-DO-192T	4.520547945	0	0.215587901	0.00617131	0.667576053	low
S98W-DO-193T	2.794520548	0	0.776037012	0.079449741	0.109430936	low
S04R-DO-198T	2.876712329	0	0.075813271	0.329518597	1.544726063	high
S06R-DO-200T	2.794520548	0	0.173846491	0.003627772	0.76635579	low
S07R-DO-201T	3.616438356	1	0.157516164	0.153314412	0.959821983	low
S08R-DO-202T	4.684931507	0	0.118059965	0	0.921324441	low
S09R-DO-203T	4.438356164	0	0.233601087	0.034534411	0.648762148	low

For Review Only

S10R-D0-204T	1.068493151	1	0.211067781	0.277825774	0.922789402	low
S11R-D0-205T	0.410958904	0	0.551106074	0.044501613	0.224701326	low
S12R-D0-206T	1.068493151	1	0.125751399	0.024094193	0.922617094	low
S14R-D0-208T	2.794520548	0	0.574701234	0.129811642	0.228609273	low
S18R-D0-212T	2.465753425	0	0.653221572	0.088956469	0.16743657	low
S22R-D0-216T	2.465753425	0	0.145627245	0	0.839469179	low
S23R-D0-217T	3.287671233	0	0.136239642	0.02323092	0.889656148	low
S26R-D0-220T	0.164383562	1	0.383673552	0.849765128	0.986669202	low
S27R-D0-221T	4.602739726	0	0.534326902	0.035942886	0.235493717	low
S29R-D0-223T	1.315068493	0	0	0.034790706	1.427650774	high
S30R-D0-224T	1.561643836	0	1.386672463	0.636007763	0.026215842	low
S37R-D0-231T	2.383561644	0	0.967809229	0.075993864	0.057060815	low
S39R-D0-233T	1.397260274	0	0.090407142	0.199890032	1.269196525	low
S46R-D0-240T	0.493150685	1	0.564035198	0.26772419	0.277168972	low
TCGA-DD-AAE3	1.550684932	0	0	0.031502632	1.422329938	high
TCGA-DD-A4NS	6.728767123	1	0.036520973	0.237759613	1.58924712	high
TCGA-DD-AADL	1.742465753	0	0	0.169074266	1.662833702	high
TCGA-ED-A7XP	1.095890411	0	0.009232238	0.335125225	1.946302688	high
TCGA-DD-A3A5	8.561643836	1	0	0.032913716	1.424610952	high
TCGA-RC-A7SK	1.293150685	0	0	0.063240389	1.474527863	high
TCGA-DD-A73D	1.898630137	0	0	0.036971953	1.431191494	high
TCGA-DD-AAVP	7.539726027	0	0	0	1.372346186	low
TCGA-DD-A4NB	2.709589041	0	0.019236534	0.089934581	1.424365623	high
TCGA-ED-A5KG	2.339726027	0	0.011072123	0.026387535	1.362223131	low
TCGA-DD-AADO	1.24109589	0	0	0	1.372346186	low
TCGA-LG-A9QD	1.002739726	0	0	0.00638393	1.382331319	low
TCGA-DD-AACA	6.304109589	0	0.005678616	0.023599608	1.382862596	low
TCGA-2Y-A9H3	4.153424658	0	0	0.02447309	1.411020997	high
TCGA-CC-A5UE	0.745205479	1	0.038793917	0.264650041	1.626004599	high
TCGA-CC-5259	0.684931507	0	0	0.034943093	1.427897851	high
TCGA-FV-A23B	5.073972603	1	0.006882885	0.048758112	1.417169047	high
TCGA-DD-AACO	5.139726027	0	0	0.013514552	1.393570275	high
TCGA-ZP-A9D2	2.095890411	1	0.016178507	0.088203768	1.436316979	high
TCGA-G3-AAV3	1.128767123	0	0	0.080876759	1.504357315	high
TCGA-K7-AAU7	0.983561644	0	0	0.228405967	1.778731555	high
TCGA-MI-A75I	1.726027397	0	0	0.036055654	1.429703039	high
TCGA-DD-AADV	1.57260274	0	0	0.142317756	1.613068721	high
TCGA-DD-AACG	1.284931507	1	0	0.079336259	1.501727897	high
TCGA-DD-AADC	1.164383562	1	0.060083073	0.094812371	1.247827934	low
TCGA-DD-AAEH	2.147945205	0	0.021725761	0.034548215	1.326346497	low
TCGA-DD-AAE9	1.978082192	0	0.017800275	0.02832857	1.33457654	low
TCGA-BC-A10S	3.898630137	1	0	0.023730991	1.409832387	high
TCGA-G3-A3CK	1.602739726	0	0	0.100930189	1.539008727	high
TCGA-RC-A7SF	1.58630137	0	0	0.03404864	1.426448209	high
TCGA-DD-AACD	1.043835616	1	0	0.009047222	1.386518432	high
TCGA-2Y-A9H0	10.06849315	0	0.027183386	0.45380264	2.096176592	high
TCGA-DD-A3A6	8.926027397	1	0	0	1.372346186	low
TCGA-3K-AAZ8	1.084931507	0	0	0.113143794	1.560503212	high
TCGA-MI-A75H	2.046575342	0	0	0.035464261	1.42874319	high

For Review Only

TCGA-ZP-A9CY	2.142465753	0	0.017990044	0.021525182	1.323457458	low
TCGA-EP-A2KB	1.632876712	1	0	0.103669296	1.543803327	high
TCGA-CC-A8HU	0.942465753	1	0	0.166383169	1.657759806	high
TCGA-DD-AADF	0.315068493	1	0	0.02645676	1.414203134	high
TCGA-DD-AACH	0.534246575	1	0	0.891607075	3.777393381	high
TCGA-BC-A10X	2.109589041	1	0.006601945	0.056991393	1.431838381	high
TCGA-CC-5262	0.282191781	1	0	0.107501867	1.550537038	high
TCGA-G3-A7M6	1.731506849	0	0.06721657	1.524038594	6.174097955	high
TCGA-G3-A7M8	1.178082192	0	0	0.035360809	1.428575349	high
TCGA-CC-A7IG	0.819178082	1	0	0.070652122	1.486991033	high
TCGA-DD-A3A4	1.676712329	1	0	0.037332765	1.43177803	high
TCGA-2Y-A9H5	1.520547945	1	0.009604507	0.037976919	1.387123707	high
TCGA-DD-A114	3.147945205	1	0	0.144828346	1.6176742	high
TCGA-BC-A110	5.797260274	1	0	0.012272249	1.391605662	high
TCGA-FV-A3I0	2.323287671	0	0	0.067466547	1.481621485	high
TCGA-UB-A7ME	1.331506849	0	0.049863964	0.483251562	2.007736521	high
TCGA-DD-AADU	1.517808219	0	0.010427386	0.00833389	1.337486743	low
TCGA-2Y-A9HB	0.712328767	0	0.008429323	0.026757489	1.37500569	low
TCGA-DD-A1EK	1.528767123	1	0	0.034957752	1.427921621	high
TCGA-DD-AAE1	1.512328767	0	0	0.289971824	1.907540892	high
TCGA-BC-A10U	2.293150685	1	0	0.051299607	1.454668221	high
TCGA-DD-AACV	4.194520548	0	0	0.455666259	2.302460074	high
TCGA-FV-A3R3	1.002739726	1	0	0.052753031	1.457071153	high
TCGA-DD-AACT	4.279452055	0	0	0.027007892	1.415088515	high
TCGA-WX-AA44	1.684931507	0	0	0.035306763	1.428487674	high
TCGA-DD-A1EH	4.095890411	0	0.013871266	0.107231557	1.479164489	high
TCGA-KR-A7K0	0.178082192	1	0	0.023224773	1.40902216	high
TCGA-DD-AAE4	1.665753425	0	0	0.02068506	1.404964242	high
TCGA-DD-AAEG	1.969863014	0	0.007576311	0.012082746	1.35618035	low
TCGA-CC-A9FW	0.679452055	0	0	0.471284556	2.343661257	high
TCGA-G3-A3CG	1.843835616	0	0	0.078942969	1.501057341	high
TCGA-DD-AAD2	1.802739726	0	0	0.019487277	1.403054499	high
TCGA-DD-AADN	2.460273973	0	0	0	1.372346186	low
TCGA-EP-A12J	1.561643836	0	0	0.022491846	1.407849897	high
TCGA-DD-AAED	2.090410959	0	0.014335779	0.034112567	1.35917162	low
TCGA-DD-AADJ	2.920547945	0	0	0	1.372346186	low
TCGA-XR-A8TF	1.898630137	1	0.020437839	0.064300967	1.377902444	low
TCGA-DD-A3A3	1.465753425	1	0	0.129341924	1.589473769	high
TCGA-DD-AAE7	1.764383562	0	0.014814554	0.058270273	1.394719163	high
TCGA-HP-A5N0	2.060273973	1	0.019149692	0.037985195	1.3431609	low
TCGA-DD-A3A2	5.838356164	1	0.008278622	0.032778461	1.385143773	low
TCGA-DD-AAEE	2.219178082	0	0.016191789	0.051101859	1.376995963	low
TCGA-UB-AA0U	0.895890411	0	0.028112849	0.015036398	1.269614582	low
TCGA-CC-A3M9	0.821917808	1	0	0.074450153	1.493418362	high
TCGA-DD-AADY	1.520547945	0	0	0.160714738	1.647122926	high
TCGA-2Y-A9GZ	2.323287671	1	0.009338542	0.221525101	1.710127618	high
TCGA-KR-A7K7	2.605479452	0	0	0.05009506	1.452679757	high
TCGA-BC-4072	4.082191781	1	0.018286319	0.033892524	1.340834312	low
TCGA-DD-AAVU	6.032876712	0	0.010304096	0.008235282	1.337893584	low

For Review Only

TCGA-BC-A8YO	1.539726027	0	0	0.55192555	2.568419075	high
TCGA-EP-A2KA	1.717808219	1	0	0.056048286	1.462533881	high
TCGA-DD-AACQ	1.183561644	1	0	0.040569946	1.437051154	high
TCGA-DD-AADI	2.97260274	0	0	0.018332772	1.401216216	high
TCGA-CC-A5UD	0.832876712	1	0.010587658	0.378531377	2.035311792	high
TCGA-BC-A10T	2.293150685	1	0.006553387	0.096377588	1.497579596	high
TCGA-XR-A8TE	2.534246575	0	0	0.054802143	1.460465677	high
TCGA-DD-AADD	3.37260274	0	0	0.13857151	1.606220897	high
TCGA-DD-A1EF	1.079452055	1	0	0.237073437	1.796325721	high
TCGA-G3-A3CJ	1.62739726	0	0	0.020832601	1.405199662	high
TCGA-DD-AAVR	6.884931507	0	0.028938859	0.030796156	1.288942796	low
TCGA-DD-AAVQ	7.473972603	0	0	0.078538532	1.500368094	high
TCGA-G3-A3CI	0.493150685	0	0	0.015433626	1.396610604	high
TCGA-ED-A805	1.112328767	0	0	0.032671163	1.424218605	high
TCGA-BC-A5W4	1.498630137	1	0.0114192	0.036167401	1.375823763	low
TCGA-2Y-A9GY	2.073972603	1	0	0.323135529	1.980750375	high
TCGA-UB-A7MD	0.142465753	1	0	0.094507082	1.527823876	high
TCGA-DD-A4NF	2.580821918	0	0.008779499	0.041593407	1.396715926	high
TCGA-DD-A113	6.643835616	0	0.013373092	0.010690379	1.327803744	low
TCGA-DD-AAW0	5.520547945	0	0	0.014226263	1.394697043	high
TCGA-DD-A1EI	0.501369863	0	0.02633651	0.21184479	1.59712488	high
TCGA-CC-A7IF	1.778082192	1	0.028613069	0.007672883	1.256918442	low
TCGA-2Y-A9GW	3.482191781	1	0.010322686	0.048807723	1.40089021	high
TCGA-DD-AAE0	1.520547945	0	0	0.505094586	2.435395447	high
TCGA-RC-A7S9	1.753424658	0	0	0.061185439	1.471090903	high
TCGA-DD-A1EG	3.75890411	1	0.014702455	0.040727249	1.36772599	low
TCGA-2Y-A9GU	5.312328767	0	0.010675401	0.058692538	1.415018609	high
TCGA-ED-A66Y	0.810958904	1	0	0.245641933	1.813890034	high
TCGA-YA-A8S7	1.128767123	1	0	0.104090616	1.544542141	high
TCGA-ED-A627	1.15890411	0	0.018512216	0.07254537	1.399932373	high
TCGA-DD-A115	6.964383562	1	0.011126187	0.111538785	1.500252803	high
TCGA-DD-A73C	1.920547945	0	0.016606126	0.0198712	1.327158513	low
TCGA-G3-AAV0	1.304109589	0	0	0.008467373	1.385605739	low
TCGA-ZP-A9D4	1.082191781	0	0.051360574	0.013852633	1.172227236	low
TCGA-ED-A806	0.153424658	1	0	0.013993695	1.394328746	high
TCGA-DD-AACJ	5.75890411	0	0	0.015068337	1.396031376	high
TCGA-DD-AAE6	0.38630137	0	0	0.078474507	1.500259012	high
TCGA-5C-A9VG	0.898630137	0	0.009661749	0.068049881	1.435036236	high
TCGA-DD-AADS	1.298630137	0	0	0.038505463	1.433686028	high
TCGA-DD-A4NV	6.569863014	0	0.039425402	0.050190815	1.271826178	low
TCGA-DD-A73E	0.120547945	0	0.010604555	0.122184843	1.521176268	high
TCGA-DD-AAEA	1.575342466	0	0.01956136	0.454899797	2.153482064	high
TCGA-HP-A5MZ	0.249315068	1	0.017196189	0.027370581	1.335846271	low
TCGA-G3-A25T	4.254794521	0	0	0.019114413	1.402460537	high
TCGA-BC-A112	0.419178082	1	0.009723448	0.05353731	1.411285975	high
TCGA-DD-A73A	1.994520548	0	0.010716488	0	1.323596353	low
TCGA-BC-4073	2.326027397	0	0.021083038	0.066299459	1.378029052	low
TCGA-DD-A4NJ	2.542465753	0	0	0.091270086	1.522217989	high
TCGA-ED-A7PY	1.068493151	0	0	0.01171294	1.390722058	high

For Review Only

TCGA-DD-A4NQ	1.021917808	1	0	0.010019776	1.388050598	high
TCGA-BD-A3ER	3.054794521	0	0	0.015240165	1.396303809	high
TCGA-DD-A1EE	0.956164384	1	0	0	1.372346186	low
TCGA-CC-A5UC	0.950684932	1	0	0.009770617	1.387657911	high
TCGA-EP-A3JL	0.830136986	0	0.009553505	0.030300493	1.375320899	low
TCGA-G3-A25Y	1.238356164	1	0.013074401	0.120680882	1.505974206	high
TCGA-DD-AADQ	1.194520548	0	0.01922496	0	1.286127222	low
TCGA-5C-A9VH	0.882191781	0	0.033234617	0.128370325	1.419251052	high
TCGA-UB-AA0V	0.860273973	0	0.05086566	0.070603779	1.252351223	low
TCGA-CC-A9FS	0.578082192	0	0	0.021833322	1.40679747	high
TCGA-G3-AAV2	1.019178082	0	0	0.007259561	1.383706551	low
TCGA-DD-A4NK	3.315068493	1	0.030927642	0.008298436	1.248023994	low
TCGA-DD-AACU	4.293150685	0	0	0.29279753	1.913671798	high
TCGA-G3-A5SL	1.701369863	0	0	0.024246862	1.410658544	high
TCGA-DD-A1EB	5.526027397	0	0.008632908	0.034168897	1.385674705	low
TCGA-BC-A10Z	0.093150685	1	0.020553405	0.256781821	1.713870592	high
TCGA-BC-A69H	1.216438356	0	0	0.016042887	1.397577224	high
TCGA-G3-A7M5	1.224657534	0	0.009246703	0.036575437	1.386591287	high
TCGA-BC-A10Y	1.947945205	1	0	0.212724836	1.747337061	high
TCGA-DD-A4NP	9.063013699	0	0.022612786	0.027047849	1.311166132	low
TCGA-BC-A10Q	3.109589041	1	0	0.05971138	1.468630434	high
TCGA-G3-AAV6	0.178082192	1	0	0.058372664	1.466399444	high
TCGA-G3-A5SI	2.104109589	1	0	0.008807382	1.386140845	high
TCGA-2Y-A9H2	4.742465753	0	0.010387401	0.073051473	1.439679866	high
TCGA-DD-AAE2	1.747945205	0	0	0.036514615	1.430448391	high
TCGA-DD-A4NL	4.687671233	0	0.041104749	0.00830119	1.205887503	low
TCGA-ES-A2HT	1.2	1	0	0.01185268	1.390942769	high
TCGA-QA-A7B7	0.257534247	0	0.075468254	0.20909572	1.348858097	low
TCGA-MR-A520	0.62739726	0	0.01786504	0	1.292043952	low
TCGA-ZS-A9CG	0.934246575	0	0	0.081919371	1.506139519	high
TCGA-DD-A73F	2.97260274	0	0	0.100800384	1.538781881	high
TCGA-DD-AADP	1.254794521	0	0.009706669	0.140699616	1.558213237	high
TCGA-GJ-A6C0	0.084931507	1	0.008175158	0.166765183	1.613343886	high
TCGA-G3-AAV1	0.983561644	1	0.008460441	0.117098721	1.523400012	high
TCGA-CC-5258	0.353424658	1	0	0.193872524	1.710326335	high
TCGA-FV-A2QR	1.591780822	1	0	0.015226989	1.396282916	high
TCGA-DD-AACB	6.367123288	0	0	0.495510158	2.40903206	high

For Review Only

TIDE score data for the entire cohort.

Patient	TIDE	Riskgroup
S01R-D0-195T-01-12R-A22L-7	3.1	low
S01W-D0-096T-01-12R-A22L-7	2.4	low
S02R-D0-196T-01-12R-A22L-7	2.09	low
S02W-D0-097T-01-12R-A22L-7	2.01	low
S03R-D0-197T-01-12R-A22L-7	1.91	low
S03W-D0-098T-01-12R-A22L-7	1.83	low
S04R-D0-198T-01-12R-A22L-7	1.82	high
S04W-D0-099T-01-12R-A22L-7	1.76	low
S05Q-D0-001T-01-12R-A22L-7	1.7	low
S05R-D0-199T-01-12R-A22L-7	1.69	low
S05W-D0-100T-01-12R-A22L-7	1.63	low
S06Q-D0-002T-01-12R-A22L-7	1.6	low
S06R-D0-200T-01-12R-A22L-7	1.57	low
S06W-D0-101T-01-12R-A22L-7	1.56	low
S07Q-D0-003T-01-12R-A22L-7	1.55	low
S07R-D0-201T-01-12R-A22L-7	1.5	low
S08Q-D0-004T-01-12R-A22L-7	1.45	low
S08R-D0-202T-01-12R-A22L-7	1.45	low
S08W-D0-103T-01-12R-A22L-7	1.45	low
S09Q-D0-005T-01-12R-A22L-7	1.43	low
S09R-D0-203T-01-12R-A22L-7	1.43	low
S09W-D0-104T-01-12R-A22L-7	1.43	low
S10Q-D0-006T-01-12R-A22L-7	1.4	low
S10R-D0-204T-01-12R-A22L-7	1.38	low
S10W-D0-105T-01-12R-A22L-7	1.37	low
S11R-D0-205T-01-12R-A22L-7	1.36	low
S11W-D0-106T-01-12R-A22L-7	1.36	low
S12Q-D0-008T-01-12R-A22L-7	1.36	high
S12R-D0-206T-01-12R-A22L-7	1.35	low
S12W-D0-107T-01-12R-A22L-7	1.33	high
S13Q-D0-009T-01-12R-A22L-7	1.31	high
S13R-D0-207T-01-12R-A22L-7	1.27	low
S13W-D0-108T-01-12R-A22L-7	1.26	low
S14Q-D0-010T-01-12R-A22L-7	1.24	low
S14R-D0-208T-01-12R-A22L-7	1.22	low
S14W-D0-109T-01-12R-A22L-7	1.22	low
S15Q-D0-011T-01-12R-A22L-7	1.22	low
S15R-D0-209T-01-12R-A22L-7	1.21	low
S15W-D0-110T-01-12R-A22L-7	1.18	high
S16Q-D0-012T-01-12R-A22L-7	1.18	low
S16R-D0-210T-01-12R-A22L-7	1.18	low
S16W-D0-111T-01-12R-A22L-7	1.17	low
S17Q-D0-013T-01-12R-A22L-7	1.17	low
S17R-D0-211T-01-12R-A22L-7	1.16	low
S17W-D0-112T-01-12R-A22L-7	1.15	low
S18Q-D0-014T-01-12R-A22L-7	1.15	low
S18R-D0-212T-01-12R-A22L-7	1.14	low

For Review Only

S18W-DO-113T-01-12R-A22L-7	1.14	low
S19Q-DO-015T-01-12R-A22L-7	1.14	low
S19R-DO-213T-01-12R-A22L-7	1.14	low
S19W-DO-114T-01-12R-A22L-7	1.14	low
S20Q-DO-016T-01-12R-A22L-7	1.13	low
S20R-DO-214T-01-12R-A22L-7	1.12	high
S21Q-DO-017T-01-12R-A22L-7	1.11	low
S21R-DO-215T-01-12R-A22L-7	1.11	low
S21W-DO-116T-01-12R-A22L-7	1.1	low
S22R-DO-216T-01-12R-A22L-7	1.1	low
S22W-DO-117T-01-12R-A22L-7	1.1	low
S23Q-DO-019T-01-12R-A22L-7	1.08	high
S23R-DO-217T-01-12R-A22L-7	1.08	low
S23W-DO-118T-01-12R-A22L-7	1.08	low
S24Q-DO-020T-01-12R-A22L-7	1.08	low
S24R-DO-218T-01-12R-A22L-7	1.07	low
S24W-DO-119T-01-12R-A22L-7	1.07	low
S25Q-DO-021T-01-12R-A22L-7	1.05	low
S25R-DO-219T-01-12R-A22L-7	1.04	low
S25W-DO-120T-01-12R-A22L-7	1.04	low
S26Q-DO-022T-01-12R-A22L-7	1.04	high
S26R-DO-220T-01-12R-A22L-7	1.04	low
S26W-DO-121T-01-12R-A22L-7	1.04	low
S27Q-DO-023T-01-12R-A22L-7	1.03	low
S27R-DO-221T-01-12R-A22L-7	1.03	low
S27W-DO-122T-01-12R-A22L-7	1.03	high
S28Q-DO-024T-01-12R-A22L-7	1.02	low
S28R-DO-222T-01-12R-A22L-7	1.02	low
S28W-DO-123T-01-12R-A22L-7	1.01	high
S29Q-DO-025T-01-12R-A22L-7	1.01	low
S29R-DO-223T-01-12R-A22L-7	1	high
S29W-DO-124T-01-12R-A22L-7	0.99	low
S30Q-DO-026T-01-12R-A22L-7	0.99	low
S30R-DO-224T-01-12R-A22L-7	0.98	low
S30W-DO-125T-01-12R-A22L-7	0.97	low
S31Q-DO-027T-01-12R-A22L-7	0.97	low
S31R-DO-225T-01-12R-A22L-7	0.97	low
S31W-DO-126T-01-12R-A22L-7	0.96	low
S32Q-DO-028T-01-12R-A22L-7	0.96	low
S32R-DO-226T-01-12R-A22L-7	0.96	low
S32W-DO-127T-01-12R-A22L-7	0.96	low
S33Q-DO-029T-01-12R-A22L-7	0.96	high
S33W-DO-128T-01-12R-A22L-7	0.95	low
S34Q-DO-030T-01-12R-A22L-7	0.95	low
S34R-DO-228T-01-12R-A22L-7	0.93	low
S34W-DO-129T-01-12R-A22L-7	0.93	low
S35Q-DO-031T-01-12R-A22L-7	0.93	low
S35R-DO-229T-01-12R-A22L-7	0.91	low
S35W-DO-130T-01-12R-A22L-7	0.9	high

For Review Only

S36Q-DO-032T-01-12R-A22L-7	0.89	high
S36R-DO-230T-01-12R-A22L-7	0.89	high
S37Q-DO-033T-01-12R-A22L-7	0.88	low
S37R-DO-231T-01-12R-A22L-7	0.87	low
S37W-DO-132T-01-12R-A22L-7	0.87	low
S38Q-DO-034T-01-12R-A22L-7	0.87	low
S38R-DO-232T-01-12R-A22L-7	0.86	low
S39Q-DO-035T-01-12R-A22L-7	0.86	low
S39R-DO-233T-01-12R-A22L-7	0.86	low
S39W-DO-134T-01-12R-A22L-7	0.84	low
S40W-DO-135T-01-12R-A22L-7	0.84	low
S41Q-DO-037T-01-12R-A22L-7	0.84	low
S41R-DO-235T-01-12R-A22L-7	0.84	low
S41W-DO-136T-01-12R-A22L-7	0.83	low
S42Q-DO-038T-01-12R-A22L-7	0.83	low
S42R-DO-236T-01-12R-A22L-7	0.82	low
S42W-DO-137T-01-12R-A22L-7	0.82	low
S43Q-DO-039T-01-12R-A22L-7	0.82	low
S43R-DO-237T-01-12R-A22L-7	0.82	low
S43W-DO-138T-01-12R-A22L-7	0.81	low
S44W-DO-139T-01-12R-A22L-7	0.8	low
S45Q-DO-041T-01-12R-A22L-7	0.8	low
S45R-DO-239T-01-12R-A22L-7	0.8	high
S45W-DO-140T-01-12R-A22L-7	0.79	low
S46Q-DO-042T-01-12R-A22L-7	0.79	high
S46R-DO-240T-01-12R-A22L-7	0.78	low
S46W-DO-141T-01-12R-A22L-7	0.78	low
S47Q-DO-043T-01-12R-A22L-7	0.78	low
S47W-DO-142T-01-12R-A22L-7	0.78	low
S48Q-DO-044T-01-12R-A22L-7	0.78	low
S49Q-DO-045T-01-12R-A22L-7	0.75	low
S49W-DO-144T-01-12R-A22L-7	0.75	low
S50Q-DO-046T-01-12R-A22L-7	0.74	low
S51Q-DO-047T-01-12R-A22L-7	0.74	low
S51W-DO-146T-01-12R-A22L-7	0.74	low
S52Q-DO-048T-01-12R-A22L-7	0.73	low
S52W-DO-147T-01-12R-A22L-7	0.73	low
S53Q-DO-049T-01-12R-A22L-7	0.73	low
S53W-DO-148T-01-12R-A22L-7	0.73	high
S54Q-DO-050T-01-12R-A22L-7	0.73	low
S54W-DO-149T-01-12R-A22L-7	0.72	low
S55Q-DO-051T-01-12R-A22L-7	0.72	low
S55W-DO-150T-01-12R-A22L-7	0.72	low
S56Q-DO-052T-01-12R-A22L-7	0.71	low
S56W-DO-151T-01-12R-A22L-7	0.71	low
S57Q-DO-053T-01-12R-A22L-7	0.71	high
S57W-DO-152T-01-12R-A22L-7	0.71	low
S58Q-DO-054T-01-12R-A22L-7	0.7	low
S58W-DO-153T-01-12R-A22L-7	0.7	low

For Review Only

S60Q-DO-056T-01-12R-A22L-7	0.69	low
S60W-DO-155T-01-12R-A22L-7	0.69	high
S61Q-DO-057T-01-12R-A22L-7	0.68	low
S61W-DO-156T-01-12R-A22L-7	0.68	low
S62Q-DO-058T-01-12R-A22L-7	0.68	low
S62W-DO-157T-01-12R-A22L-7	0.67	low
S63Q-DO-059T-01-12R-A22L-7	0.67	low
S63W-DO-158T-01-12R-A22L-7	0.66	low
S64Q-DO-060T-01-12R-A22L-7	0.66	low
S64W-DO-159T-01-12R-A22L-7	0.66	high
S65Q-DO-061T-01-12R-A22L-7	0.65	low
S65W-DO-160T-01-12R-A22L-7	0.65	high
S66Q-DO-062T-01-12R-A22L-7	0.65	low
S67Q-DO-063T-01-12R-A22L-7	0.64	high
S67W-DO-162T-01-12R-A22L-7	0.63	low
S68Q-DO-064T-01-12R-A22L-7	0.63	low
S68W-DO-163T-01-12R-A22L-7	0.63	low
S69Q-DO-065T-01-12R-A22L-7	0.63	low
S69W-DO-164T-01-12R-A22L-7	0.63	low
S70Q-DO-066T-01-12R-A22L-7	0.62	high
S70W-DO-165T-01-12R-A22L-7	0.62	low
S71Q-DO-067T-01-12R-A22L-7	0.62	low
S71W-DO-166T-01-12R-A22L-7	0.62	high
S72Q-DO-068T-01-12R-A22L-7	0.62	low
S72W-DO-167T-01-12R-A22L-7	0.62	low
S73Q-DO-069T-01-12R-A22L-7	0.62	low
S73W-DO-168T-01-12R-A22L-7	0.62	low
S74Q-DO-070T-01-12R-A22L-7	0.61	low
S74W-DO-169T-01-12R-A22L-7	0.61	low
S75Q-DO-071T-01-12R-A22L-7	0.61	low
S75W-DO-170T-01-12R-A22L-7	0.61	low
S76Q-DO-072T-01-12R-A22L-7	0.6	low
S76W-DO-171T-01-12R-A22L-7	0.6	low
S77Q-DO-073T-01-12R-A22L-7	0.59	high
S77W-DO-172T-01-12R-A22L-7	0.59	low
S78Q-DO-074T-01-12R-A22L-7	0.59	low
S78W-DO-173T-01-12R-A22L-7	0.58	low
S79W-DO-174T-01-12R-A22L-7	0.57	low
S80Q-DO-076T-01-12R-A22L-7	0.57	low
S80W-DO-175T-01-12R-A22L-7	0.56	low
S81Q-DO-077T-01-12R-A22L-7	0.56	high
S81W-DO-176T-01-12R-A22L-7	0.56	low
S82Q-DO-078T-01-12R-A22L-7	0.55	low
S82W-DO-177T-01-12R-A22L-7	0.55	high
S83Q-DO-079T-01-12R-A22L-7	0.55	low
S83W-DO-178T-01-12R-A22L-7	0.55	low
S84Q-DO-080T-01-12R-A22L-7	0.55	low
S84W-DO-179T-01-12R-A22L-7	0.54	low
S85Q-DO-081T-01-12R-A22L-7	0.53	low

For Review Only

S85W-D0-180T-01-12R-A22L-7	0.53	low
S86Q-D0-082T-01-12R-A22L-7	0.52	low
S86W-D0-181T-01-12R-A22L-7	0.52	low
S87Q-D0-083T-01-12R-A22L-7	0.52	low
S88Q-D0-084T-01-12R-A22L-7	0.51	low
S88W-D0-183T-01-12R-A22L-7	0.51	low
S89Q-D0-085T-01-12R-A22L-7	0.51	low
S90Q-D0-086T-01-12R-A22L-7	0.5	low
S90W-D0-185T-01-12R-A22L-7	0.5	low
S91Q-D0-087T-01-12R-A22L-7	0.5	low
S91W-D0-186T-01-12R-A22L-7	0.5	low
S92Q-D0-088T-01-12R-A22L-7	0.49	high
S92W-D0-187T-01-12R-A22L-7	0.49	high
S93Q-D0-089T-01-12R-A22L-7	0.49	low
S93W-D0-188T-01-12R-A22L-7	0.49	high
S94Q-D0-090T-01-12R-A22L-7	0.49	low
S94W-D0-189T-01-12R-A22L-7	0.48	low
S95Q-D0-091T-01-12R-A22L-7	0.48	low
S95W-D0-190T-01-12R-A22L-7	0.47	low
S96W-D0-191T-01-12R-A22L-7	0.46	low
S97Q-D0-093T-01-12R-A22L-7	0.46	low
S97W-D0-192T-01-12R-A22L-7	0.46	low
S98Q-D0-094T-01-12R-A22L-7	0.46	low
S98W-D0-193T-01-12R-A22L-7	0.45	low
S99W-D0-194T-01-12R-A22L-7	0.44	high
TCGA-2Y-A9GS-01A-12R-A38B-7	0.44	high
TCGA-2Y-A9GT-01A-11R-A38B-7	0.44	high
TCGA-2Y-A9GU-01A-11R-A38B-7	0.43	high
TCGA-2Y-A9GV-01A-11R-A38B-7	0.43	low
TCGA-2Y-A9GW-01A-11R-A38B-7	0.43	high
TCGA-2Y-A9GX-01A-11R-A38B-7	0.43	low
TCGA-2Y-A9GY-01A-11R-A38B-7	0.42	high
TCGA-2Y-A9GZ-01A-11R-A39D-7	0.42	high
TCGA-2Y-A9H0-01A-11R-A38B-7	0.42	high
TCGA-2Y-A9H1-01A-11R-A38B-7	0.41	low
TCGA-2Y-A9H2-01A-12R-A38B-7	0.41	high
TCGA-2Y-A9H3-01A-11R-A38B-7	0.41	high
TCGA-2Y-A9H4-01A-11R-A38B-7	0.41	high
TCGA-2Y-A9H5-01A-11R-A38B-7	0.41	high
TCGA-2Y-A9H6-01A-11R-A39D-7	0.4	high
TCGA-2Y-A9H7-01A-11R-A39D-7	0.4	low
TCGA-2Y-A9H8-01A-11R-A39D-7	0.39	high
TCGA-2Y-A9H9-01A-21R-A39D-7	0.39	low
TCGA-2Y-A9HA-01A-11R-A39D-7	0.39	high
TCGA-2Y-A9HB-01A-11R-A39D-7	0.39	low
TCGA-3K-AAZ8-01A-12R-A39D-7	0.39	high
TCGA-4R-AA8I-01A-11R-A38B-7	0.38	high
TCGA-5C-A9VG-01A-11R-A37K-7	0.38	high
TCGA-5C-A9VH-01A-11R-A37K-7	0.38	high

For Review Only

TCGA-5R-AA1C-01A-11R-A41C-7	0.38	high
TCGA-5R-AA1D-01A-11R-A38B-7	0.37	low
TCGA-5R-AAAM-01A-12R-A41C-7	0.37	high
TCGA-BC-4073-01B-02R-A131-7	-0.8	low
TCGA-BC-4072-01B-11R-A155-7	-0.81	low
TCGA-BC-A10Q-01A-11R-A131-7	0.36	high
TCGA-BC-A10R-01A-11R-A131-7	0.36	high
TCGA-BC-A10S-01A-22R-A131-7	0.36	high
TCGA-BC-A10T-01A-11R-A131-7	0.36	high
TCGA-BC-A10U-01A-11R-A131-7	0.35	high
TCGA-BC-A10W-01A-11R-A131-7	0.35	high
TCGA-BC-A10X-01A-11R-A131-7	0.35	high
TCGA-BC-A10Y-01A-11R-A131-7	0.35	high
TCGA-BC-A10Z-01A-11R-A131-7	0.34	high
TCGA-BC-A110-01A-11R-A131-7	0.34	high
TCGA-BC-A112-01A-11R-A131-7	0.34	high
TCGA-BC-A216-01A-11R-A155-7	0.34	high
TCGA-BC-A217-01A-11R-A155-7	0.34	high
TCGA-BC-A3KG-01A-11R-A213-7	0.33	high
TCGA-BC-A5W4-01A-11R-A28V-7	0.33	low
TCGA-BC-A69H-01A-11R-A311-7	0.32	high
TCGA-BC-A69I-01A-11R-A311-7	0.32	low
TCGA-BC-A8YO-01A-11R-A37K-7	0.32	high
TCGA-BD-A2L6-01A-11R-A213-7	0.31	high
TCGA-BD-A3EP-01A-11R-A22L-7	0.31	high
TCGA-BD-A3ER-01A-11R-A213-7	0.31	high
TCGA-CC-5258-01A-01R-A131-7	0.3	high
TCGA-CC-5259-01A-31R-A213-7	0.3	high
TCGA-CC-5260-01A-01R-A131-7	0.3	high
TCGA-CC-5261-01A-01R-A131-7	0.29	high
TCGA-CC-5262-01A-01R-A131-7	0.29	high
TCGA-CC-5263-01A-01R-A131-7	0.28	high
TCGA-CC-5264-01A-01R-A131-7	0.28	high
TCGA-CC-A123-01A-11R-A131-7	0.28	low
TCGA-CC-A1HT-01A-11R-A131-7	0.28	high
TCGA-CC-A3M9-01A-11R-A213-7	0.28	high
TCGA-CC-A3MA-01A-11R-A213-7	0.28	high
TCGA-CC-A3MB-01A-11R-A213-7	0.27	high
TCGA-CC-A3MC-01A-11R-A22L-7	0.26	high
TCGA-CC-A5UC-01A-11R-A28V-7	0.26	high
TCGA-CC-A5UD-01A-11R-A28V-7	0.26	high
TCGA-CC-A5UE-01A-11R-A28V-7	0.26	high
TCGA-CC-A7IE-01A-21R-A38B-7	0.25	high
TCGA-CC-A7IF-01A-11R-A33J-7	0.25	low
TCGA-CC-A7IG-01A-11R-A33J-7	0.25	high
TCGA-CC-A7IH-01A-11R-A33J-7	0.24	high
TCGA-CC-A7II-01A-11R-A33J-7	0.24	high
TCGA-CC-A7IJ-01A-11R-A33R-7	0.24	high
TCGA-CC-A7IK-01A-12R-A33R-7	0.24	high

For Review Only

TCGA-CC-A7IL-01A-11R-A33R-7	0.24	low
TCGA-CC-A8HS-01A-11R-A36F-7	0.24	low
TCGA-CC-A8HT-01A-11R-A36F-7	0.24	high
TCGA-CC-A8HU-01A-11R-A36F-7	0.23	high
TCGA-CC-A8HV-01A-11R-A36F-7	0.22	high
TCGA-CC-A9FS-01A-11R-A37K-7	0.22	high
TCGA-CC-A9FW-01A-11R-A37K-7	0.21	high
TCGA-DD-A113-01A-11R-A131-7	0.21	low
TCGA-DD-A114-01A-11R-A131-7	0.21	high
TCGA-DD-A115-01A-11R-A131-7	0.21	high
TCGA-DD-A116-01A-11R-A131-7	0.2	low
TCGA-DD-A118-01A-11R-A131-7	0.2	high
TCGA-DD-A119-01A-11R-A131-7	0.2	high
TCGA-DD-A11A-01A-11R-A131-7	0.2	high
TCGA-DD-A11C-01A-11R-A131-7	0.19	high
TCGA-DD-A11D-01A-11R-A131-7	0.19	low
TCGA-DD-A1EA-01A-11R-A131-7	0.19	high
TCGA-DD-A1EB-01A-11R-A131-7	0.19	low
TCGA-DD-A1EC-01A-21R-A131-7	0.19	high
TCGA-DD-A1ED-01A-11R-A155-7	0.19	low
TCGA-DD-A1EE-01A-11R-A131-7	0.19	low
TCGA-DD-A1EF-01A-11R-A131-7	0.18	high
TCGA-DD-A1EG-01A-11R-A213-7	0.18	low
TCGA-DD-A1EH-01A-11R-A131-7	0.18	high
TCGA-DD-A1EI-01A-11R-A131-7	0.18	high
TCGA-DD-A1EJ-01A-11R-A155-7	0.17	high
TCGA-DD-A1EK-01A-11R-A213-7	0.17	high
TCGA-DD-A1EL-01A-11R-A155-7	0.17	high
TCGA-DD-A39V-01A-11R-A213-7	0.17	low
TCGA-DD-A39W-01A-11R-A213-7	0.16	high
TCGA-DD-A39X-01A-11R-A213-7	0.16	high
TCGA-DD-A39Y-01A-11R-A213-7	0.16	high
TCGA-DD-A39Z-01A-11R-A213-7	0.16	low
TCGA-DD-A3A1-01A-11R-A213-7	0.16	high
TCGA-DD-A3A2-01A-11R-A213-7	0.16	low
TCGA-DD-A3A3-01A-11R-A22L-7	0.16	high
TCGA-DD-A3A4-01A-11R-A22L-7	0.15	high
TCGA-DD-A3A5-01A-11R-A22L-7	0.15	high
TCGA-DD-A3A6-01A-11R-A22L-7	0.15	low
TCGA-DD-A3A7-01A-11R-A22L-7	0.15	high
TCGA-DD-A3A9-01A-11R-A266-7	0.14	high
TCGA-DD-A4NA-01A-11R-A266-7	0.14	high
TCGA-DD-A4NB-01A-12R-A266-7	0.13	high
TCGA-DD-A4ND-01A-11R-A266-7	0.13	low
TCGA-DD-A4NE-01A-11R-A27V-7	0.13	high
TCGA-DD-A4NF-01A-11R-A27V-7	0.13	high
TCGA-DD-A4NG-01A-11R-A27V-7	0.13	high
TCGA-DD-A4NH-01A-11R-A27V-7	0.13	high
TCGA-DD-A4NI-01A-11R-A27V-7	0.12	low

For Review Only

TCGA-DD-A4NJ-01A-11R-A27V-7	0.12	high
TCGA-DD-A4NK-01A-11R-A28V-7	0.12	low
TCGA-DD-A4NL-01A-11R-A28V-7	0.12	low
TCGA-DD-A4NN-01A-11R-A28V-7	0.12	high
TCGA-DD-A4NO-01A-11R-A28V-7	0.11	low
TCGA-DD-A4NP-01A-11R-A28V-7	0.11	low
TCGA-DD-A4NQ-01A-21R-A28V-7	0.11	high
TCGA-DD-A4NS-01A-11R-A311-7	0.11	high
TCGA-DD-A4NV-01A-11R-A311-7	0.1	low
TCGA-DD-A73A-01A-12R-A320-7	0.1	low
TCGA-DD-A73B-01A-12R-A320-7	0.09	low
TCGA-DD-A73C-01A-12R-A33J-7	0.09	low
TCGA-DD-A73D-01A-12R-A320-7	0.08	high
TCGA-DD-A73E-01A-12R-A320-7	0.08	high
TCGA-DD-A73F-01A-11R-A320-7	0.07	high
TCGA-DD-A73G-01A-22R-A320-7	0.07	high
TCGA-DD-AA3A-01A-11R-A37K-7	0.06	high
TCGA-DD-AAC9-01A-11R-A41C-7	0.05	high
TCGA-DD-AACA-01A-11R-A41C-7	0.05	low
TCGA-DD-AACB-01A-11R-A41C-7	0.05	high
TCGA-DD-AACC-01A-11R-A41C-7	0.05	low
TCGA-DD-AACD-01A-11R-A41C-7	0.04	high
TCGA-DD-AACE-01A-11R-A41C-7	0.04	low
TCGA-DD-AACF-01A-11R-A41C-7	0.04	low
TCGA-DD-AACG-01A-11R-A41C-7	0.04	high
TCGA-DD-AACH-01A-11R-A41C-7	0.04	high
TCGA-DD-AACI-01A-11R-A41C-7	0.04	high
TCGA-DD-AACJ-01A-11R-A41C-7	0.04	high
TCGA-DD-AACL-01A-11R-A41C-7	0.03	high
TCGA-DD-AACN-01A-11R-A41C-7	0.03	low
TCGA-DD-AACO-01A-11R-A41C-7	0.03	high
TCGA-DD-AACP-01A-11R-A41C-7	0.03	high
TCGA-DD-AACQ-01A-11R-A41C-7	0.03	high
TCGA-DD-AACS-01A-11R-A41C-7	0.02	low
TCGA-DD-AACT-01A-11R-A41C-7	0.02	high
TCGA-DD-AACU-01A-11R-A41C-7	0.02	high
TCGA-DD-AACV-01A-11R-A41C-7	0.01	high
TCGA-DD-AACW-01A-11R-A41C-7	0.01	low
TCGA-DD-AACX-01A-11R-A41C-7	0.01	high
TCGA-DD-AACY-01A-11R-A41C-7	0.01	high
TCGA-DD-AACZ-01A-11R-A41C-7	0.01	high
TCGA-DD-AAD0-01A-11R-A41C-7	0.01	high
TCGA-DD-AAD1-01A-11R-A41C-7	0.01	high
TCGA-DD-AAD2-01A-11R-A41C-7	0	high
TCGA-DD-AAD3-01A-11R-A41C-7	0	high
TCGA-DD-AAD5-01A-11R-A41C-7	-0.01	high
TCGA-DD-AAD6-01A-11R-A41C-7	-0.01	high
TCGA-DD-AAD8-01A-11R-A41C-7	-0.01	low
TCGA-DD-AADA-01A-11R-A41C-7	-0.03	high

For Review Only

TCGA-DD-AADB-01A-11R-A41C-7	-0.03	low
TCGA-DD-AADC-01A-11R-A41C-7	-0.03	low
TCGA-DD-AADD-01A-11R-A41C-7	-0.03	high
TCGA-DD-AADF-01A-11R-A41C-7	-0.04	high
TCGA-DD-AADG-01A-11R-A41C-7	-0.04	high
TCGA-DD-AADI-01A-11R-A41C-7	-0.04	high
TCGA-DD-AADJ-01A-11R-A41C-7	-0.05	low
TCGA-DD-AADK-01A-11R-A41C-7	-0.05	low
TCGA-DD-AADL-01A-11R-A41C-7	-0.05	high
TCGA-DD-AADN-01A-11R-A41C-7	-0.05	low
TCGA-DD-AADO-01A-11R-A41C-7	-0.06	low
TCGA-DD-AADP-01A-11R-A39D-7	-0.06	high
TCGA-DD-AADQ-01A-11R-A41C-7	-0.06	low
TCGA-DD-AADR-01A-11R-A41C-7	-0.06	high
TCGA-DD-AADS-01A-11R-A41C-7	-0.06	high
TCGA-DD-AADU-01A-11R-A41C-7	-0.07	low
TCGA-DD-AADV-01A-11R-A39D-7	-0.07	high
TCGA-DD-AADW-01A-11R-A39D-7	-0.07	high
TCGA-DD-AADY-01A-11R-A41C-7	-0.07	high
TCGA-DD-AAE0-01A-11R-A41C-7	-0.07	high
TCGA-DD-AAE1-01A-11R-A41C-7	-0.08	high
TCGA-DD-AAE2-01A-11R-A41C-7	-0.08	high
TCGA-DD-AAE3-01A-11R-A41C-7	-0.08	high
TCGA-DD-AAE4-01A-11R-A41C-7	-0.08	high
TCGA-DD-AAE6-01A-11R-A41C-7	-0.08	high
TCGA-DD-AAE7-01A-11R-A41C-7	-0.08	high
TCGA-DD-AAE9-01A-11R-A41C-7	-0.08	low
TCGA-DD-AAEA-01A-11R-A41C-7	-0.08	high
TCGA-DD-AAEB-01A-11R-A41C-7	-0.09	high
TCGA-DD-AAED-01A-12R-A41C-7	-0.09	low
TCGA-DD-AAEE-01A-11R-A41C-7	-0.09	low
TCGA-DD-AAEG-01A-11R-A39D-7	-0.09	low
TCGA-DD-AAEH-01A-11R-A41C-7	-0.1	low
TCGA-DD-AAEI-01A-11R-A41C-7	-0.1	high
TCGA-DD-AAEK-01A-11R-A41C-7	-0.12	low
TCGA-DD-AAVP-01A-11R-A41C-7	-0.12	low
TCGA-DD-AAVQ-01A-11R-A41C-7	-0.12	high
TCGA-DD-AAVR-01A-11R-A41C-7	-0.12	low
TCGA-DD-AAVS-01A-11R-A41C-7	-0.13	high
TCGA-DD-AAVU-01A-11R-A41C-7	-0.13	low
TCGA-DD-AAVV-01A-11R-A41C-7	-0.13	high
TCGA-DD-AAVW-01A-11R-A41C-7	-0.14	high
TCGA-DD-AAVX-01A-11R-A41C-7	-0.14	high
TCGA-DD-AAVY-01A-11R-A41C-7	-0.14	low
TCGA-DD-AAVZ-01A-11R-A41C-7	-0.15	high
TCGA-DD-AAW0-01A-11R-A41C-7	-0.15	high
TCGA-DD-AAW1-01A-11R-A41C-7	-0.15	high
TCGA-DD-AAW2-01A-11R-A41C-7	-0.16	low
TCGA-DD-AAW3-01A-11R-A41C-7	-0.16	high

For Review Only

TCGA-ED-A459-01A-11R-A266-7	-0.16	high
TCGA-ED-A4XI-01A-11R-A266-7	-0.17	low
TCGA-ED-A5KG-01A-11R-A27V-7	-0.17	low
TCGA-ED-A627-01A-12R-A311-7	-0.17	high
TCGA-ED-A66X-01A-11R-A311-7	-0.18	high
TCGA-ED-A66Y-01A-11R-A311-7	-0.18	high
TCGA-ED-A7PY-01A-11R-A33R-7	-0.18	high
TCGA-ED-A7XO-01A-11R-A352-7	-0.19	high
TCGA-ED-A7XP-01A-11R-A352-7	-0.2	high
TCGA-ED-A82E-01A-11R-A352-7	-0.2	high
TCGA-ED-A8O5-01A-11R-A36F-7	-0.2	high
TCGA-ED-A8O6-01A-11R-A36F-7	-0.21	high
TCGA-EP-A12J-01A-11R-A131-7	-0.21	high
TCGA-EP-A26S-01A-11R-A16W-7	-0.22	high
TCGA-EP-A2KA-01A-11R-A180-7	-0.22	high
TCGA-EP-A2KB-01A-11R-A180-7	-0.23	high
TCGA-EP-A3JL-01A-11R-A213-7	-0.23	low
TCGA-EP-A3RK-01A-11R-A22L-7	-0.23	high
TCGA-ES-A2HS-01A-11R-A180-7	-0.23	low
TCGA-ES-A2HT-01A-12R-A180-7	-0.23	high
TCGA-FV-A23B-01A-11R-A16W-7	-0.25	high
TCGA-FV-A2QQ-01A-11R-A22L-7	-0.25	high
TCGA-FV-A2QR-01A-11R-A213-7	-0.25	high
TCGA-FV-A3I0-01A-11R-A22L-7	-0.26	high
TCGA-FV-A3I1-01A-11R-A22L-7	-0.26	high
TCGA-FV-A3R2-01A-11R-A22L-7	-0.26	low
TCGA-FV-A3R3-01A-11R-A22L-7	-0.27	high
TCGA-FV-A4ZP-01A-12R-A266-7	-0.27	high
TCGA-G3-A25S-01A-11R-A16W-7	-0.28	high
TCGA-G3-A25T-01A-11R-A16W-7	-0.28	high
TCGA-G3-A25U-01A-11R-A16W-7	-0.28	low
TCGA-G3-A25V-01A-11R-A16W-7	-0.29	high
TCGA-G3-A25X-01A-11R-A16W-7	-0.3	high
TCGA-G3-A25Y-01A-11R-A16W-7	-0.3	high
TCGA-G3-A25Z-01A-11R-A16W-7	-0.33	high
TCGA-G3-A3CG-01A-11R-A213-7	-0.34	high
TCGA-G3-A3CH-01A-11R-A22L-7	-0.34	high
TCGA-G3-A3CI-01A-11R-A213-7	-0.34	high
TCGA-G3-A3CJ-01A-11R-A213-7	-0.34	high
TCGA-G3-A3CK-01A-11R-A213-7	-0.35	high
TCGA-G3-A5SI-01A-31R-A27V-7	-0.37	high
TCGA-G3-A5SJ-01A-11R-A27V-7	-0.37	high
TCGA-G3-A5SK-01A-11R-A27V-7	-0.37	low
TCGA-G3-A5SL-01A-11R-A27V-7	-0.38	high
TCGA-G3-A5SM-01A-12R-A28V-7	-0.39	high
TCGA-G3-A6UC-01A-21R-A33J-7	-0.4	high
TCGA-G3-A7M5-01A-11R-A33R-7	-0.4	high
TCGA-G3-A7M6-01A-11R-A33R-7	-0.41	high
TCGA-G3-A7M7-01A-12R-A352-7	-0.41	low

For Review Only

TCGA-G3-A7M8-01A-11R-A33R-7	-0.42	high
TCGA-G3-A7M9-01A-23R-A352-7	-0.42	high
TCGA-G3-AAUZ-01A-11R-A38B-7	-0.43	high
TCGA-G3-AAV0-01A-11R-A37K-7	-0.43	low
TCGA-G3-AAV1-01A-11R-A38B-7	-0.43	high
TCGA-G3-AAV2-01A-11R-A37K-7	-0.43	low
TCGA-G3-AAV3-01A-11R-A37K-7	-0.43	high
TCGA-G3-AAV5-01A-11R-A37K-7	-0.44	high
TCGA-G3-AAV6-01A-21R-A37K-7	-0.44	high
TCGA-G3-AAV7-01A-11R-A38B-7	-0.44	high
TCGA-GJ-A3OU-01A-31R-A38B-7	-0.44	high
TCGA-GJ-A6C0-01A-12R-A311-7	-0.46	high
TCGA-GJ-A9DB-01A-11R-A37K-7	-0.46	high
TCGA-HP-A5MZ-01A-21R-A27V-7	-0.47	low
TCGA-HP-A5N0-01A-11R-A28V-7	-0.48	low
TCGA-K7-A5RF-01A-11R-A28V-7	-0.48	low
TCGA-K7-A5RG-01A-11R-A28V-7	-0.48	high
TCGA-K7-A6G5-01A-11R-A311-7	-0.48	low
TCGA-K7-AAU7-01A-11R-A38B-7	-0.48	high
TCGA-KR-A7K0-01A-12R-A33R-7	-0.49	high
TCGA-KR-A7K2-01A-12R-A33R-7	-0.5	high
TCGA-KR-A7K7-01A-11R-A33J-7	-0.5	high
TCGA-KR-A7K8-01A-11R-A33J-7	-0.51	high
TCGA-LG-A6GG-01A-11R-A311-7	-0.51	high
TCGA-LG-A9QC-01A-11R-A37K-7	-0.51	high
TCGA-LG-A9QD-01A-11R-A38B-7	-0.53	low
TCGA-MI-A75C-01A-11R-A320-7	-0.53	high
TCGA-MI-A75E-01A-11R-A320-7	-0.53	high
TCGA-MI-A75G-01A-11R-A320-7	-0.54	low
TCGA-MI-A75H-01A-11R-A320-7	-0.54	high
TCGA-MI-A75I-01A-11R-A320-7	-0.55	high
TCGA-MR-A520-01A-11R-A266-7	-0.55	low
TCGA-MR-A8JO-01A-12R-A36F-7	-0.55	low
TCGA-NI-A4U2-01A-11R-A28V-7	-0.55	high
TCGA-NI-A8LF-01A-11R-A36F-7	-0.55	low
TCGA-O8-A75V-01A-11R-A320-7	-0.55	high
TCGA-PD-A5DF-01A-11R-A27V-7	-0.59	high
TCGA-QA-A7B7-01A-11R-A320-7	-0.6	low
TCGA-RC-A7S9-01A-11R-A33R-7	-0.65	high
TCGA-RC-A7SB-01A-21R-A352-7	-0.65	high
TCGA-RC-A7SF-01A-11R-A352-7	-0.65	high
TCGA-RC-A7SH-01A-11R-A38B-7	-0.65	low
TCGA-RC-A7SK-01A-11R-A352-7	-0.66	high
TCGA-RG-A7D4-01A-12R-A33R-7	-0.66	high
TCGA-UB-A7MA-01A-11R-A33R-7	-0.68	low
TCGA-UB-A7MB-01A-11R-A33R-7	-0.69	high
TCGA-UB-A7MC-01A-11R-A33R-7	-0.69	low
TCGA-UB-A7MD-01A-12R-A352-7	-0.7	high
TCGA-UB-A7ME-01A-11R-A33J-7	-0.7	high

For Review Only

TCGA-UB-A7MF-01A-11R-A33J-7	-0.71	high
TCGA-UB-AA0U-01A-11R-A38B-7	-0.72	low
TCGA-UB-AA0V-01A-11R-A38B-7	-0.72	low
TCGA-WJ-A86L-01A-12R-A39D-7	-0.72	high
TCGA-WQ-A9G7-01A-11R-A37K-7	-0.73	high
TCGA-WQ-AB4B-01A-11R-A41C-7	-0.73	high
TCGA-WX-AA44-01A-11R-A39D-7	-0.73	high
TCGA-WX-AA46-01A-11R-A39D-7	-0.73	low
TCGA-WX-AA47-01A-11R-A39D-7	-0.74	high
TCGA-XR-A8TC-01A-11R-A36F-7	-0.74	low
TCGA-XR-A8TD-01A-12R-A39D-7	-0.74	high
TCGA-XR-A8TE-01A-11R-A36F-7	-0.74	high
TCGA-XR-A8TF-01A-11R-A36F-7	-0.75	low
TCGA-XR-A8TG-01A-11R-A36F-7	-0.76	high
TCGA-YA-A8S7-01A-11R-A37K-7	-0.76	high
TCGA-ZP-A9CV-01A-11R-A38B-7	-0.77	high
TCGA-ZP-A9CY-01A-11R-A38B-7	-0.78	low
TCGA-ZP-A9CZ-01A-11R-A38B-7	-0.78	low
TCGA-ZP-A9D0-01A-11R-A37K-7	-0.78	low
TCGA-ZP-A9D2-01A-11R-A38B-7	-0.79	high
TCGA-ZP-A9D4-01A-11R-A37K-7	-0.8	low
TCGA-ZS-A9CD-01A-11R-A37K-7	-0.8	low
TCGA-ZS-A9CE-01A-11R-A37K-7	-0.8	high
TCGA-ZS-A9CF-01A-11R-A38B-7	-0.8	high
TCGA-ZS-A9CG-01A-11R-A37K-7	-2.61	high

For Review Only

TMB value data for the entire cohort.

Edited ID	TMB	Riskgroup
S01R-DO-195T	0.026315789	low
S01W-DO-096T	1.210526316	low
S02R-DO-196T	2.421052632	low
S02W-DO-097T	0.684210526	low
S03R-DO-197T	0.657894737	low
S03W-DO-098T	4.815789474	low
S04R-DO-198T	1.684210526	high
S04W-DO-099T	1.605263158	low
S05Q-DO-001T	0.605263158	low
S05R-DO-199T	0.236842105	low
S05W-DO-100T	0.157894737	low
S06Q-DO-002T	5.473684211	low
S06R-DO-200T	0.894736842	low
S06W-DO-101T	1.026315789	low
S07Q-DO-003T	0.789473684	low
S07R-DO-201T	10.63157895	low
S08Q-DO-004T	0.526315789	low
S08R-DO-202T	0.421052632	low
S08W-DO-103T	1	low
S09Q-DO-005T	3.289473684	low
S09W-DO-104T	0.921052632	low
S10Q-DO-006T	2.078947368	low
S10R-DO-204T	0.5	low
S10W-DO-105T	0.5	low
S11R-DO-205T	0.368421053	low
S11W-DO-106T	0.052631579	low
S12R-DO-206T	3.052631579	low
S12W-DO-107T	0.157894737	high
S13Q-DO-009T	0.578947368	high
S13R-DO-207T	1.210526316	low
S14Q-DO-010T	0.210526316	low
S14R-DO-208T	0.447368421	low
S14W-DO-109T	0.605263158	low
S15Q-DO-011T	0.526315789	low
S15W-DO-110T	0.631578947	high
S16Q-DO-012T	0.921052632	low
S16W-DO-111T	0.894736842	low
S17Q-DO-013T	0.947368421	low
S17R-DO-211T	0.5	low
S17W-DO-112T	0.078947368	low
S18Q-DO-014T	0.236842105	low
S18W-DO-113T	0.342105263	low
S19Q-DO-015T	0.578947368	low
S19W-DO-114T	1.026315789	low
S20Q-DO-016T	0.894736842	low
S20R-DO-214T	0.973684211	high
S21Q-DO-017T	0.026315789	low

For Review Only

S21R-DO-215T	3.842105263	low
S21W-DO-116T	0.368421053	low
S22R-DO-216T	0.868421053	low
S22W-DO-117T	1.710526316	low
S23Q-DO-019T	1.157894737	high
S23W-DO-118T	0.657894737	low
S24Q-DO-020T	0.684210526	low
S24R-DO-218T	0.842105263	low
S24W-DO-119T	0.315789474	low
S25Q-DO-021T	2.263157895	low
S25R-DO-219T	1.421052632	low
S25W-DO-120T	0.473684211	low
S26Q-DO-022T	0.5	high
S26R-DO-220T	0.710526316	low
S26W-DO-121T	0.421052632	low
S27Q-DO-023T	0.578947368	low
S27R-DO-221T	1.605263158	low
S27W-DO-122T	1.184210526	high
S28Q-DO-024T	0.473684211	low
S28R-DO-222T	1.789473684	low
S28W-DO-123T	0.552631579	high
S29Q-DO-025T	0.210526316	low
S29W-DO-124T	0.131578947	low
S30Q-DO-026T	1.657894737	low
S30W-DO-125T	0.394736842	low
S31Q-DO-027T	0.736842105	low
S31W-DO-126T	0.342105263	low
S32Q-DO-028T	0.394736842	low
S33Q-DO-029T	0.342105263	high
S33W-DO-128T	1.131578947	low
S34Q-DO-030T	1.736842105	low
S34W-DO-129T	2.973684211	low
S35Q-DO-031T	0.421052632	low
S35W-DO-130T	0.684210526	high
S36Q-DO-032T	4.105263158	high
S37Q-DO-033T	0.473684211	low
S38Q-DO-034T	0.026315789	low
S39Q-DO-035T	0.789473684	low
S41Q-DO-037T	0.526315789	low
S42Q-DO-038T	0.315789474	low
S42W-DO-137T	0.5	low
S43Q-DO-039T	0.578947368	low
S43W-DO-138T	0.105263158	low
S44W-DO-139T	0.052631579	low
S45Q-DO-041T	0.631578947	low
S45W-DO-140T	0.868421053	low
S46Q-DO-042T	1.684210526	high
S46W-DO-141T	0.315789474	low
S47Q-DO-043T	0.394736842	low

For Review Only

S47W-DO-142T	0.184210526	low
S48Q-DO-044T	0.921052632	low
S49W-DO-144T	2.973684211	low
S50Q-DO-046T	0.552631579	low
S51Q-DO-047T	0.815789474	low
S51W-DO-146T	0.736842105	low
S52W-DO-147T	0.657894737	low
S53W-DO-148T	2.736842105	high
S54Q-DO-050T	0.894736842	low
S54W-DO-149T	0.105263158	low
S55Q-DO-051T	2.289473684	low
S55W-DO-150T	1.105263158	low
S56Q-DO-052T	1.131578947	low
S57W-DO-152T	2.5	low
S58W-DO-153T	0.236842105	low
S60Q-DO-056T	0.552631579	low
S60W-DO-155T	0.157894737	high
S61W-DO-156T	0.473684211	low
S62Q-DO-058T	2.710526316	low
S62W-DO-157T	0.473684211	low
S63Q-DO-059T	0.315789474	low
S64Q-DO-060T	0.263157895	low
S65Q-DO-061T	0.605263158	low
S66Q-DO-062T	0.763157895	low
S67Q-DO-063T	0.684210526	high
S67W-DO-162T	0.315789474	low
S68W-DO-163T	0.552631579	low
S69W-DO-164T	0.210526316	low
S70Q-DO-066T	1.210526316	high
S70W-DO-165T	0.368421053	low
S71Q-DO-067T	0.5	low
S71W-DO-166T	0.131578947	high
S72Q-DO-068T	1.368421053	low
S72W-DO-167T	0.605263158	low
S73Q-DO-069T	0.578947368	low
S73W-DO-168T	0.368421053	low
S74Q-DO-070T	1.131578947	low
S74W-DO-169T	0.552631579	low
S75W-DO-170T	0.473684211	low
S76Q-DO-072T	0.421052632	low
S77Q-DO-073T	0.5	high
S77W-DO-172T	5.815789474	low
S78Q-DO-074T	0.631578947	low
S78W-DO-173T	0.447368421	low
S79W-DO-174T	2.026315789	low
S80Q-DO-076T	0.421052632	low
S80W-DO-175T	0.552631579	low
S81Q-DO-077T	6.184210526	high
S81W-DO-176T	0.210526316	low

For Review Only

S82Q-D0-078T	1.605263158	low
S82W-D0-177T	0.131578947	high
S83Q-D0-079T	0.342105263	low
S83W-D0-178T	0.631578947	low
S84Q-D0-080T	0.421052632	low
S84W-D0-179T	0.236842105	low
S85Q-D0-081T	0.921052632	low
S85W-D0-180T	3.315789474	low
S86Q-D0-082T	0.710526316	low
S86W-D0-181T	0.526315789	low
S88W-D0-183T	0.947368421	low
S91Q-D0-087T	0.447368421	low
S91W-D0-186T	1.342105263	low
S92Q-D0-088T	2.236842105	high
S92W-D0-187T	0.342105263	high
S93Q-D0-089T	0.894736842	low
S93W-D0-188T	0.631578947	high
S94Q-D0-090T	1.105263158	low
S94W-D0-189T	1.421052632	low
S95Q-D0-091T	0.157894737	low
S95W-D0-190T	0.447368421	low
S96W-D0-191T	0.236842105	low
S97Q-D0-093T	0.289473684	low
S97W-D0-192T	0.736842105	low
S98Q-D0-094T	0.5	low
S98W-D0-193T	5.552631579	low
S99W-D0-194T	1.289473684	high
TCGA-2Y-A9GS	1.394736842	high
TCGA-2Y-A9GT	1.973684211	high
TCGA-2Y-A9GU	2.894736842	high
TCGA-2Y-A9GV	1.342105263	low
TCGA-2Y-A9GW	1.605263158	high
TCGA-2Y-A9GX	0.815789474	low
TCGA-2Y-A9GY	1.605263158	high
TCGA-2Y-A9GZ	1.973684211	high
TCGA-2Y-A9H0	1.710526316	high
TCGA-2Y-A9H1	2.868421053	low
TCGA-2Y-A9H2	1.157894737	high
TCGA-2Y-A9H3	2.684210526	high
TCGA-2Y-A9H4	1.473684211	high
TCGA-2Y-A9H5	1.631578947	high
TCGA-2Y-A9H6	1.105263158	high
TCGA-2Y-A9H7	1.868421053	low
TCGA-2Y-A9H8	1.5	high
TCGA-2Y-A9H9	2.736842105	low
TCGA-2Y-A9HA	2.973684211	high
TCGA-2Y-A9HB	1.473684211	low
TCGA-3K-AAZ8	2.815789474	high
TCGA-4R-AA8I	24.44736842	high

For Review Only

TCGA-5C-A9VG	2.789473684	high
TCGA-5C-A9VH	2.236842105	high
TCGA-5R-AA1C	2.026315789	high
TCGA-5R-AA1D	0.210526316	low
TCGA-5R-AAAM	1.342105263	high
TCGA-BC-4073	2.842105263	low
TCGA-BC-A10Q	0.736842105	high
TCGA-BC-A10R	2.315789474	high
TCGA-BC-A10T	1.552631579	high
TCGA-BC-A10U	3.026315789	high
TCGA-BC-A10W	2.184210526	high
TCGA-BC-A10Y	1.289473684	high
TCGA-BC-A10Z	4.052631579	high
TCGA-BC-A216	1.263157895	high
TCGA-BC-A217	2.157894737	high
TCGA-BC-A5W4	1.526315789	low
TCGA-BC-A69H	2.736842105	high
TCGA-BC-A8YO	1.526315789	high
TCGA-BD-A2L6	1.789473684	high
TCGA-BD-A3EP	2.236842105	high
TCGA-BD-A3ER	0.973684211	high
TCGA-CC-5258	2.894736842	high
TCGA-CC-5259	2.921052632	high
TCGA-CC-5260	1.078947368	high
TCGA-CC-5262	2.5	high
TCGA-CC-5263	2.710526316	high
TCGA-CC-5264	2.157894737	high
TCGA-CC-A123	1.736842105	low
TCGA-CC-A1HT	1.631578947	high
TCGA-CC-A3M9	2.473684211	high
TCGA-CC-A3MA	0.947368421	high
TCGA-CC-A3MB	1.342105263	high
TCGA-CC-A3MC	2.105263158	high
TCGA-CC-A5UC	1.105263158	high
TCGA-CC-A5UD	4.552631579	high
TCGA-CC-A5UE	3.131578947	high
TCGA-CC-A7IE	3.421052632	high
TCGA-CC-A7IF	2.605263158	low
TCGA-CC-A7IG	3.052631579	high
TCGA-CC-A7IH	16.13157895	high
TCGA-CC-A7II	3.526315789	high
TCGA-CC-A7IJ	3.157894737	high
TCGA-CC-A7IK	6.736842105	high
TCGA-CC-A7IL	2.263157895	low
TCGA-CC-A8HS	1.105263158	low
TCGA-CC-A8HT	4.368421053	high
TCGA-CC-A8HU	1.921052632	high
TCGA-CC-A8HV	3.184210526	high
TCGA-CC-A9FS	2.289473684	high

For Review Only

TCGA-CC-A9FW	3.342105263	high
TCGA-DD-A113	2.5	low
TCGA-DD-A114	2.105263158	high
TCGA-DD-A115	1.631578947	high
TCGA-DD-A116	2.131578947	low
TCGA-DD-A118	2.605263158	high
TCGA-DD-A119	1.157894737	high
TCGA-DD-A11A	2.368421053	high
TCGA-DD-A11C	2.105263158	high
TCGA-DD-A11D	2.578947368	low
TCGA-DD-A1EA	1.842105263	high
TCGA-DD-A1EB	3	low
TCGA-DD-A1EC	0.526315789	high
TCGA-DD-A1ED	0.368421053	low
TCGA-DD-A1EE	7.210526316	low
TCGA-DD-A1EF	1.868421053	high
TCGA-DD-A1EH	0.684210526	high
TCGA-DD-A1EI	1.210526316	high
TCGA-DD-A1EJ	1.236842105	high
TCGA-DD-A1EK	1.184210526	high
TCGA-DD-A1EL	1.947368421	high
TCGA-DD-A39V	1.5	low
TCGA-DD-A39W	0.736842105	high
TCGA-DD-A39X	1.342105263	high
TCGA-DD-A39Z	1.526315789	low
TCGA-DD-A3A2	1.447368421	low
TCGA-DD-A3A3	1.184210526	high
TCGA-DD-A3A4	0.710526316	high
TCGA-DD-A3A5	1.342105263	high
TCGA-DD-A3A6	0.342105263	low
TCGA-DD-A3A7	2.447368421	high
TCGA-DD-A3A9	6.657894737	high
TCGA-DD-A4NA	0.657894737	high
TCGA-DD-A4NB	0.447368421	high
TCGA-DD-A4ND	1.184210526	low
TCGA-DD-A4NE	1.210526316	high
TCGA-DD-A4NF	2.578947368	high
TCGA-DD-A4NG	1.631578947	high
TCGA-DD-A4NH	1.157894737	high
TCGA-DD-A4NI	2.578947368	low
TCGA-DD-A4NJ	1.631578947	high
TCGA-DD-A4NK	1.526315789	low
TCGA-DD-A4NL	0.447368421	low
TCGA-DD-A4NN	1.210526316	high
TCGA-DD-A4NO	1.131578947	low
TCGA-DD-A4NP	0.447368421	low
TCGA-DD-A4NQ	1.973684211	high
TCGA-DD-A4NS	0.342105263	high
TCGA-DD-A4NV	2.342105263	low

For Review Only

TCGA-DD-A73A	1.552631579	low
TCGA-DD-A73B	1.789473684	low
TCGA-DD-A73C	1.657894737	low
TCGA-DD-A73D	1.421052632	high
TCGA-DD-A73E	2.473684211	high
TCGA-DD-A73F	1.210526316	high
TCGA-DD-A73G	2.342105263	high
TCGA-DD-AA3A	0.868421053	high
TCGA-DD-AAC9	1.578947368	high
TCGA-DD-AACA	2.421052632	low
TCGA-DD-AACB	2.184210526	high
TCGA-DD-AACC	1.447368421	low
TCGA-DD-AACD	1.973684211	high
TCGA-DD-AACE	1.578947368	low
TCGA-DD-AACF	2.815789474	low
TCGA-DD-AACG	2.210526316	high
TCGA-DD-AACH	2.105263158	high
TCGA-DD-AACI	6.684210526	high
TCGA-DD-AACJ	2.078947368	high
TCGA-DD-AACL	6.368421053	high
TCGA-DD-AACN	0.947368421	low
TCGA-DD-AACO	1	high
TCGA-DD-AACP	3.289473684	high
TCGA-DD-AACQ	4.447368421	high
TCGA-DD-AACS	1.105263158	low
TCGA-DD-AACT	4	high
TCGA-DD-AACU	2.263157895	high
TCGA-DD-AACV	2.263157895	high
TCGA-DD-AACW	1.210526316	low
TCGA-DD-AACX	2.736842105	high
TCGA-DD-AACY	1.605263158	high
TCGA-DD-AACZ	3.552631579	high
TCGA-DD-AAD0	2.289473684	high
TCGA-DD-AAD1	2.263157895	high
TCGA-DD-AAD2	1.657894737	high
TCGA-DD-AAD3	1.447368421	high
TCGA-DD-AAD5	2.710526316	high
TCGA-DD-AAD6	2.184210526	high
TCGA-DD-AAD8	2.368421053	low
TCGA-DD-AADA	2.552631579	high
TCGA-DD-AADB	2.5	low
TCGA-DD-AADC	1.789473684	low
TCGA-DD-AADD	1.921052632	high
TCGA-DD-AADF	4.763157895	high
TCGA-DD-AADG	3.131578947	high
TCGA-DD-AADI	1.842105263	high
TCGA-DD-AADJ	1.394736842	low
TCGA-DD-AADK	1.210526316	low
TCGA-DD-AADL	2.763157895	high

For Review Only

TCGA-DD-AADN	2.289473684	low
TCGA-DD-AADO	4.447368421	low
TCGA-DD-AADP	2.210526316	high
TCGA-DD-AADQ	2.605263158	low
TCGA-DD-AADR	2	high
TCGA-DD-AADS	3	high
TCGA-DD-AADU	2.026315789	low
TCGA-DD-AADV	1.868421053	high
TCGA-DD-AADW	0.736842105	high
TCGA-DD-AADY	1.078947368	high
TCGA-DD-AAE0	1.368421053	high
TCGA-DD-AAE1	1.105263158	high
TCGA-DD-AAE2	1.578947368	high
TCGA-DD-AAE3	3.421052632	high
TCGA-DD-AAE4	1.263157895	high
TCGA-DD-AAE6	1.710526316	high
TCGA-DD-AAE7	4.736842105	high
TCGA-DD-AAE9	2.342105263	low
TCGA-DD-AAEA	4	high
TCGA-DD-AAEB	2.947368421	high
TCGA-DD-AAED	1.5	low
TCGA-DD-AAEE	1.605263158	low
TCGA-DD-AAEG	1.736842105	low
TCGA-DD-AAEH	1.921052632	low
TCGA-DD-AAEI	2.736842105	high
TCGA-DD-AAEK	1.631578947	low
TCGA-DD-AAVP	1.631578947	low
TCGA-DD-AAVQ	0.947368421	high
TCGA-DD-AAVR	1.342105263	low
TCGA-DD-AAVS	1.131578947	high
TCGA-DD-AAVU	1.184210526	low
TCGA-DD-AAVV	1.921052632	high
TCGA-DD-AAVW	0.763157895	high
TCGA-DD-AAVX	1.5	high
TCGA-DD-AAVY	2.052631579	low
TCGA-DD-AAVZ	0.921052632	high
TCGA-DD-AAW0	2.026315789	high
TCGA-DD-AAW1	2.342105263	high
TCGA-DD-AAW2	2.552631579	low
TCGA-DD-AAW3	1.973684211	high
TCGA-ED-A459	3.052631579	high
TCGA-ED-A4XI	1.947368421	low
TCGA-ED-A66X	0.815789474	high
TCGA-ED-A66Y	2.052631579	high
TCGA-ED-A7PY	0.605263158	high
TCGA-ED-A7XO	1.263157895	high
TCGA-ED-A7XP	1.421052632	high
TCGA-ED-A82E	0.605263158	high
TCGA-ED-A8O5	2.657894737	high

For Review Only

TCGA-ED-A806	1.552631579	high
TCGA-EP-A12J	1.052631579	high
TCGA-EP-A26S	1.921052632	high
TCGA-EP-A2KA	2.315789474	high
TCGA-EP-A2KB	1.736842105	high
TCGA-EP-A3JL	1.947368421	low
TCGA-EP-A3RK	1.157894737	high
TCGA-ES-A2HS	1.315789474	low
TCGA-ES-A2HT	1.447368421	high
TCGA-FV-A23B	1.894736842	high
TCGA-FV-A2QQ	2.763157895	high
TCGA-FV-A2QR	2.421052632	high
TCGA-FV-A3I0	1.815789474	high
TCGA-FV-A3I1	1.5	high
TCGA-FV-A3R2	1.842105263	low
TCGA-FV-A3R3	0.842105263	high
TCGA-FV-A4ZP	2.289473684	high
TCGA-G3-A25S	3	high
TCGA-G3-A25T	1.026315789	high
TCGA-G3-A25U	1.5	low
TCGA-G3-A25V	1.078947368	high
TCGA-G3-A25Y	1.526315789	high
TCGA-G3-A25Z	1.605263158	high
TCGA-G3-A3CG	2	high
TCGA-G3-A3CH	0.631578947	high
TCGA-G3-A3CI	0.342105263	high
TCGA-G3-A3CK	4.605263158	high
TCGA-G3-A5SI	0.605263158	high
TCGA-G3-A5SJ	2.315789474	high
TCGA-G3-A5SK	1.710526316	low
TCGA-G3-A5SL	2.605263158	high
TCGA-G3-A5SM	2.263157895	high
TCGA-G3-A6UC	2.684210526	high
TCGA-G3-A7M5	4.131578947	high
TCGA-G3-A7M6	1.763157895	high
TCGA-G3-A7M7	1.421052632	low
TCGA-G3-A7M8	0.421052632	high
TCGA-G3-A7M9	2.710526316	high
TCGA-G3-AAUZ	1.815789474	high
TCGA-G3-AAV0	3.605263158	low
TCGA-G3-AAV1	1.184210526	high
TCGA-G3-AAV2	0.973684211	low
TCGA-G3-AAV3	1.921052632	high
TCGA-G3-AAV5	1.868421053	high
TCGA-G3-AAV6	1.368421053	high
TCGA-G3-AAV7	1.736842105	high
TCGA-GJ-A3OU	0.894736842	high
TCGA-GJ-A6C0	1.631578947	high
TCGA-GJ-A9DB	1.631578947	high

For Review Only

TCGA-HP-A5MZ	1	low
TCGA-HP-A5N0	2.736842105	low
TCGA-K7-A5RF	0.157894737	low
TCGA-K7-A5RG	2.368421053	high
TCGA-K7-A6G5	1.578947368	low
TCGA-K7-AAU7	1.526315789	high
TCGA-KR-A7K0	1.973684211	high
TCGA-KR-A7K2	1.131578947	high
TCGA-KR-A7K7	1.526315789	high
TCGA-KR-A7K8	0.815789474	high
TCGA-LG-A6GG	4.157894737	high
TCGA-LG-A9QC	0.973684211	high
TCGA-LG-A9QD	2.289473684	low
TCGA-MI-A75C	2.473684211	high
TCGA-MI-A75E	2.578947368	high
TCGA-MI-A75G	5.078947368	low
TCGA-MI-A75H	2.894736842	high
TCGA-MI-A75I	3.657894737	high
TCGA-MR-A520	0.315789474	low
TCGA-MR-A8JO	0.5	low
TCGA-NI-A4U2	2.342105263	high
TCGA-NI-A8LF	2.210526316	low
TCGA-O8-A75V	1.552631579	high
TCGA-PD-A5DF	0.973684211	high
TCGA-QA-A7B7	1.947368421	low
TCGA-RC-A7S9	1.289473684	high
TCGA-RC-A7SB	1.210526316	high
TCGA-RC-A7SF	1.921052632	high
TCGA-RC-A7SH	1.447368421	low
TCGA-RC-A7SK	2.763157895	high
TCGA-RG-A7D4	1.736842105	high
TCGA-UB-A7MA	1.736842105	low
TCGA-UB-A7MB	31.57894737	high
TCGA-UB-A7MC	1.421052632	low
TCGA-UB-A7MD	2.368421053	high
TCGA-UB-A7ME	1.789473684	high
TCGA-UB-A7MF	2.026315789	high
TCGA-UB-AA0U	1.789473684	low
TCGA-UB-AA0V	0.578947368	low
TCGA-WJ-A86L	3.078947368	high
TCGA-WQ-A9G7	10.02631579	high
TCGA-WQ-AB4B	1.289473684	high
TCGA-WX-AA44	1.605263158	high
TCGA-WX-AA46	0.631578947	low
TCGA-WX-AA47	0.789473684	high
TCGA-XR-A8TC	1.236842105	low
TCGA-XR-A8TD	1.263157895	high
TCGA-XR-A8TE	0.184210526	high
TCGA-XR-A8TF	3.052631579	low

For Review Only

TCGA-XR-A8TG	1.631578947	high
TCGA-YA-A8S7	1.315789474	high
TCGA-ZP-A9CV	2.210526316	high
TCGA-ZP-A9CY	1.842105263	low
TCGA-ZP-A9CZ	1.052631579	low
TCGA-ZP-A9D0	1.263157895	low
TCGA-ZP-A9D2	1.368421053	high
TCGA-ZP-A9D4	1.421052632	low
TCGA-ZS-A9CD	1.973684211	low
TCGA-ZS-A9CE	2.710526316	high
TCGA-ZS-A9CF	1.947368421	high
TCGA-ZS-A9CG	1.736842105	high

For Review Only
